# Catheter configuration for mapping micro-anatomic reentries sustaining atrial fibrillation: A simulation study

**DOI:** 10.1371/journal.pcbi.1014493

**Published:** 2026-07-24

**Authors:** Miguel Rodrigo, Giada S. Romitti, María Termenón-Rivas, Ning Li, Vadim V. Fedorov

**Affiliations:** 1 CoMMLab, Electronic Engineering & Computer Science Department, Universitat de València, València, Spain; 2 Department of Physiology & Cell Biology, Bob and Corrine Frick Center for Heart Failure and Arrhythmia, Dorothy M. Davis Heart & Lung Research Institute, The Ohio State University College of Medicine and Wexner Medical Center, Columbus, Ohio, United States of America; Nanjing University, CHINA

## Abstract

Atrial fibrillation (AF) can be sustained by intramural reentrant circuits within three-dimensional arrhythmogenic hubs formed by fibrotically-insulated myobundles. However, the efficacy of different multi-electrode mapping (MEM) to identify the micro-reentrant pathways sustaining AF remains undefined. An anisotropic atrial tissue structure (30 × 30 × 4 mm), incorporating a sub-endocardial laterally-insulated myobundle (15 × 2.5 × 1.5 mm) was simulated reflecting persistent AF conditions. Simulations included endocardial unipolar, bipolar, and omnipolar electrograms, with local activation time maps calculated for reentry visualization. N = 656 MEM configurations were evaluated, varying inter-electrode distances (1, 3, 6 and 9 mm), orientations (parallel and perpendicular), contact distances to the wall (0.25 and 1.0 mm), and electrode positions (in 1-mm increments) relative to the reentrant circuit. Conduction along the reentrant pathway was identified by electrograms within <3 mm of the micro-reentrant circuit, and confirmed by their comparison to action potential traces. However, detection on electrogram (EGM) traces was highly dependent on catheter configuration and distance to the atrial wall. Dense unipolar MEM configurations (1–6 mm spacing) facilitated pathway identification, while bipolar MEM required electrode pairs to align with the myobundle for effective detection. Omnipolar configurations offered no significant advantages over unipolar for modest inter-electrode spacings (1–6 mm) but improved detection accuracy at larger spacings (9 mm). Mapping was affected by micro-reentrant track width, though reentrant mapping still detected tracks thinner than electrode spacing. Track thickness and conduction velocity did not impair detection and sometimes improved it. Unipolar MEM configurations (1–6 mm spacing) with optimal contact enabled the detection of sub-endocardial reentry pathways sustaining AF in 50–100% of simulated cases. Combining unipolar and omnipolar mapping approaches (3 mm spacing) may enhance the detection rates of AF micro-reentry. These findings provide critical insights into optimizing MEM techniques for human AF reentrant circuit detection and may improve the efficacy of AF ablation procedures.

## Author summary

Atrial fibrillation (AF) is the most common heart rhythm disorder and is linked to a high risk of stroke and other complications. AF can be sustained by tiny electrical loops, called micro-reentrant circuits, that form within small bundles of heart muscle insulated by scar tissue. Identifying these circuits is essential because they can serve as key targets for ablation procedures, a common treatment for AF. However, current mapping techniques using multi-electrode catheters vary in their ability to detect these reentrant pathways. In our study, we used computer simulations to test different mapping strategies and electrode configurations under conditions that mimic persistent AF. We found that dense unipolar electrode arrangements with close tissue contact were most effective in identifying the reentrant pathways. While bipolar and omnipolar mapping offered some benefits in specific settings, unipolar configurations consistently provided accurate detection, when combined with optimal spacing and contact, or omnipolar mapping. These results highlight practical ways to improve mapping strategies for AF, which could lead to more effective ablation treatments and better outcomes for patients living with this complex heart rhythm disorder.

## Introduction

Atrial fibrillation (AF), a prevalent cardiac arrhythmia globally, poses significant morbidity and mortality challenges. Current treatments for AF have limited efficacy and can lead to serious side effects [1]. Primary theories regarding the maintenance mechanisms of persistent AF (perAF) revolve around either multi-wavelets propagating across the atria or localized AF drivers, electrical phenomena critical to AF perpetuation [2]. More recently, AF has been proposed to be sustained by drivers characterized by rapid reentrant electrical activity that spreads throughout the atria and can be temporally stable or unstable [3]. Targeted ablation of AF drivers reduces the ability to sustain AF and has shown promise in improving treatment efficacy and long-term outcomes compared to conventional pulmonary vein isolation [4–5]. However, guided ablation strategies going beyond pulmonary vein isolation currently lack sufficient scientific evidence to be recommended in clinical practice [6]. Accurately identifying patient-specific AF drivers poses a challenge due to the inadequate visualization of potential intramural reentrant drivers using surface-only multi-electrode mapping (MEM) [7]. This limitation has resulted in conflicting clinical mapping results and outcomes in AF driver ablation trials [2,8].

In contrast to MEM, near-infrared optical mapping (NIOM) offers high resolution and subsurface views of electrical conduction [9], enabling detailed visualization of intramural reentry as a primary mechanism of human AF drivers [9]. Recent *ex vivo* studies on human atria using transmural NIOM and contrast-enhanced magnetic resonance imaging (CE-MRI) have revealed that AF can be sustained by a limited number of intramural reentry circuits within 3D arrhythmogenic hubs comprised of fibrotically insulated myobundles [10].

However, the optimal MEM configuration needed to identify common reentrant tracks sustaining AF in clinical scenarios remain unknown. This computational work simulated human AF episodes sustained by the micro-anatomical reentrant circuit with the common track for preferential conduction within a sub-endocardial, laterally-insulated myobundles, in which different MEM configurations are tested to identify the micro-reentrant path.

## Materials and methods

### Biophysical simulation of a micro-anatomical reentry

**Fig 1:**
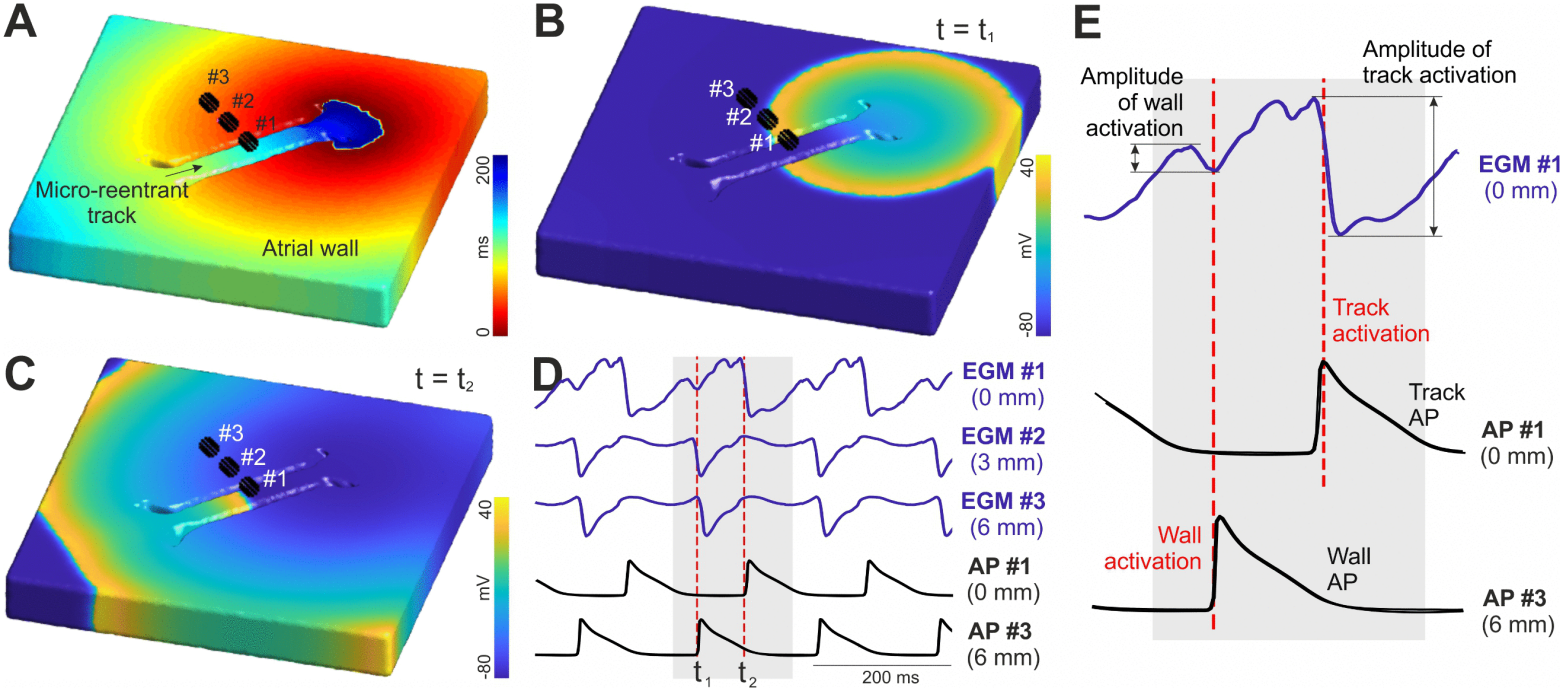
Simulation of micro-anatomical reentry. **(A)** Local activation time (LAT) map. **(B)** Transmembrane potential during initial atrial activation. **(C)** Transmembrane potential during micro-anatomical reentry activation. **(D)** Electrogram (EGM) traces (blue) recorded from electrodes positioned 0, 3, and 6 mm from the micro-anatomical reentry (panel **A)**, compared with transmembrane potentials (black) from their closest points on the micro-anatomical reentry (AP #1) and atrial wall (AP #3). **(E)** Detailed amplitude estimation for wall and track activations.

A 3D model of an atrial slab of tissue, including an insulated myobundle (336,241 nodes, 2,017,775 tetrahedral, 267 ± 53 µm inter-node distance) was used to simulate an AF pattern sustained by a micro-anatomical reentry. Based on prior *ex vivo* human studies of atrial driver regions [7,9,11–13], we constructed a simplified but representative human atrial driver-region geometry rather than a full chamber-specific reconstruction of either the right atrium (RA) or left atrium (LA). The model was designed to capture the essential 3D structural configuration shared by human atrial driver substrates, including a sub-endocardial, laterally-insulated myobundle reproducing the essential common micro-anatomic reentrant track configuration described in prior structural-functional studies. This reduced geometry was selected to preserve the key substrate features relevant to both RA and LA driver regions while allowing controlled evaluation of how electrode spacing, orientation, and wall contact influence MEM-based detection of the reentrant track. Based on the 3D structure of AF driver reentrant track revealed by *ex vivo* human heart 3D imaging [9], we constructed a 3D anatomical model consisting of a wedge of atrial wall tissue (30 × 30 × 4 mm) with a sub-endocardial, laterally-insulated myobundle (15 × 2.5 × 1.5 mm) (Fig 1), embedded in the atrial wall tissue.

Atrial electrophysiology node was simulated using the mono-domain cellular model described by Koivumäki et al. [14] and solved using a GPU-based algorithm [15]. Electrical remodeling was introduced into the cellular model to simulate chronic AF by modifying the following ionic currents: SERCA expression (-16%), PLB to SERCA ratio (+18%), SLN to SERCA ratio (-40%), maximal I_NCX_ (+50%), sensitivity of RyR to [Ca^2+^]_SR_ (+100%), conductance of I_CaL_ (-59%), conductance of I_to_ (-44%), conductance of I_Kur_ (-22%) and conductance of I_K1_ (+100%) [14]. Heterogeneity in the electrophysiological properties of sub-endocardial laterally-insulated myobundle with respect to the atrial myocardium was introduced as reduced tissue conductivity (-40% respect to atrial wall) in order to simulate slow-path conduction identified in experimental studies [16]. Anisotropy of the cardiac tissue was applied varying the longitudinal and transversal diffusion (1:1.36 ratio in conduction velocity) [17–19] respect to the fiber direction, which was constant and aligned with the laterally-insulated myobundle orientation. Diffusion tensor was calculated with its maximal component D_long_ aligned with the fiber direction and the minimal D_trans_, perpendicular to the fiber direction. The simulation was first stabilized by regular pacing from the atrial wall next to the myobundle exit site (cycle length = 200 ms, 10 beats), and the reentry was initiated by the induction of a conduction block in the myobundle exit site.

In addition to the anatomical model described above, simulations were performed on three similar models to evaluate the effect of the atrial substrate on micro-reentry detection, considering the micro-reentrant track’s anatomical configuration and electrical conduction conditions. For this purpose, the aforementioned anatomical model was used as the baseline model and various variations were created based on it. First, the thickness of the micro-reentrant path was reduced to 0.75 mm, being this width 50% of the baseline model. In the second anatomical model, the width of the micro-reentrant path was halved (to 1.25 mm) compared with the baseline model. Finally, a simulation with adjusted diffusion (-16% in the atrial wall and -20% in the micro-reentrant track respect to the baseline model) was performed to achieve similar conduction velocities in the atrial wall and the micro-reentrant track.

### Electrogram calculation

Simulated unipolar EGMs were calculated using cylindrical electrodes (0.5 mm radius, 1 mm length, similar to grid and basket-type commercial catheters) positioned on a 2D plane above the endocardial surface, using two different distances in the normal direction (0.25, 1 mm) between these electrodes and endocardial wall where the myobundle is embedded. EGMs were calculated using Eq. (1) adding all effective transmembrane contributions over the entire tissue volume:


$$\begin{array}{c} u_{e}\left(\vec{r_{e}},t\right)=-\;\frac{\text{1}}{\text{4}\pi \sigma }\int_{V}^{\;}\vec{\nabla }u_{m}\cdot \left(\frac{\vec{r}}{{\left|\vec{r}\right|}^{\text{3}}}\right)dV\;,\; \end{array}$$ (1)

where $u_{e}$ denotes the extracellular potential, σ denotes the extracellular conductivity, V denotes the total tissue volume, $\vec{\nabla }u_{m}$ denotes the gradient of the transmembrane potential and $\vec{r}$ is the vector from the active tissue dV to the recording point $\vec{r_{e}}$. The computed EGM were stored for processing at a sampling frequency of 500 Hz.

Three different EGM configurations were used: unipolar, bipolar and omnipolar EGM traces. Bipolar EGMs were calculated as the difference between unipolar EGM traces at different spacing, calculated in the same direction (0º) and perpendicular (90º) to the sub-endocardial micro-reentrant path. Omnipolar EGM traces were calculated using groups (cliques) of 4 electrodes in 2 × 2 arrangements, in which diagonal bipoles were used to construct omnipolar EGM traces [20]. Omnipolar EGMs were computed as the orthogonal transformation that maximizes the amplitude of the activation. This omnipolar disposition provides equivalent resolution to unipolar and bipolar EGM maps, and it has been defended as more robust for local activation time (LAT) estimation [20].

### Multi-electrode mapping

To evaluate the effect of the electrode distribution and location to properly map this micro-reentry, N = 656 electrode configurations were used for mapping, described on Table 1. Different inter-electrode distances (1, 3, 6 and 9 mm) were evaluated, as well as 2 different separations respect to the atrial wall (0.25 mm and 1 mm). For each configuration, the MEM catheter, defined as an initial square grid of N_x_ × N_y_ electrodes, was systematically displaced relative to the micro-reentrant pathway. The displacement consisted of a 1 mm shift from the initial position along the two axes parallel to the atrial wall, generating horizontal, vertical, and combined offsets in both directions. When the grid was displaced, electrodes falling outside the atrial geometry were excluded from the analysis, resulting in reduced/rectangular N_x_ × N_y_ grid sizes in some configurations. The simulations were divided into 254 unipolar, 292 bipolar and 110 omnipolar configurations; 328 configurations for each separation (0.25 and 1 mm); and 8, 72, 288 and 288 for each inter-electrode distance (1, 3, 6 and 9 mm).

**Table 1:** Electrode configuration. Number of simulations performed per electrode configuration. Size: unipolar electrodes per configuration (N_x_ × N_y_).

			Electrode type			
Contact	Electrode spacing	Sizes N_x_ × N_y_	Unipolar	Bipolar 0º	Bipolar 90º	Omnipolar
0.25 mm	**1 mm**	21 × 21	N = 1	N = 1	N = 1	N = 1
	**3 mm**	7 × 7, 6 × 7, 6 × 6	N = 9	N = 9	N = 9	N = 9
	**6 mm**	4 × 4, 3 × 4, 3 × 3	N = 36	N = 36	N = 36	N = 36
	**9 mm**	3 × 3, 3 × 2, 2 × 2	N = 81	N = 27	N = 27	N = 9
1 mm	**1 mm**	21 × 21	N = 1	N = 1	N = 1	N = 1
	**3 mm**	7 × 7, 6 × 7, 6 × 6	N = 9	N = 9	N = 9	N = 9
	**6 mm**	4 × 4, 3 × 4, 3 × 3	N = 36	N = 36	N = 36	N = 36
	**9 mm**	3 × 3, 3 × 2, 2 × 2	N = 81	N = 27	N = 27	N = 9

On unipolar EGM maps, LAT was calculated by identifying the activation as the instant with maximal negative derivative of voltage over time. On bipolar and omnipolar EGM traces, LAT was identified as the instant with maximal absolute amplitude. Omnipolar calculation was used also to identify conduction velocity and propagation direction.

**Fig 2:**
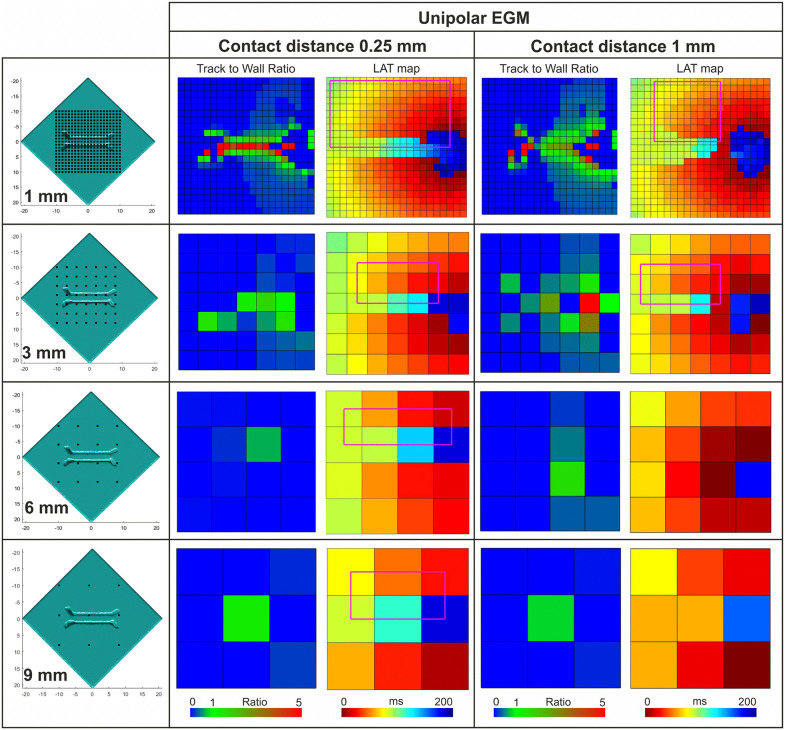
Unipolar EGM mapping of micro-anatomical reentry. Left panels illustrate the schematic EGM positions relative to the reentrant structure for each inter-electrode spacing (1, 3, 6, and 9 mm). Middle and right panels display the recorded reentry track-to-wall ratio and LAT maps, respectively, for each inter-electrode spacing (1, 3, 6, and 9 mm) and two EGM distances to the atrial wall (0.25 mm and 1 mm). Magenta rectangles on LAT maps represent the reentrant path identified, with the highest coverage of the cycle length.

LAT maps with different electrode configurations were used to evaluate the micro-reentry presence. They were automatically evaluated by identifying rectangles along the LAT map with potential reentrant LAT paths, defined in ≥4 contiguous electrodes. An intensive search for all the rectangles in the LAT was carried out, and reentrant LAT paths were considered when the LAT values of the rectangular path presented a quasi-monotonic increasing/decreasing pattern, starting from the minimal LAT, with deviations < 3 ms from monotonicity. The percentage of the reentrant sequence covering the cycle was calculated as the cycle length minus the highest increment between consecutive electrodes, in %. Micro-reentrant paths were identified in LAT maps when rectangular, reentrant paths covered >65% of the cycle length, and the rectangle with the highest coverage was selected (Fig 2). Maps with global LAT spanning <50% of the cycle length were not evaluated for reentrant paths.

Additionally, a reentrant track-to-wall EGM ratio was calculated as the ratio between the amplitude of unipolar EGM signals corresponding to myobundle activation and atrial wall activation, determined by the timing of the nearest transmembrane potential signal (Fig 1E). This metric allowed us to assess the relative contribution of the sub-endocardial, laterally-insulated myobundle to the EGM signal, where ratios > 1 indicated cases in which the myobundle contribution to the EGM was greater than that of the atrial wall.

## Results

### Electrogram traces on micro-reentrant track

Fig 1A shows the LAT map for the simulation conducted in which a micro-anatomical reentry was sustaining the AF activity. This reentrant path showed a cycle length of 174 ± 11 ms, with a conduction velocity of 17 ± 2 cm/sec along the common reentrant path through the insulated myobundle and 26 ± 6 cm/sec along the two returning paths through the surrounding atrial wall. Partial conduction block and source–sink mismatch at the exit of the micro-reentrant track produced discontinuous propagation, increasing the effective reentrant path length to ~36 mm, slightly longer than twice the physical myobundle length of 15 mm. Panels B and C illustrate the extent of tissue activation, showing that the atrial wall (Panel B) activated a larger area compared to the common reentrant path (Panel C). Consequently, atrial wall activation is expected to contribute more prominently to the unipolar EGM signals (Panel D).

In Panel D, only unipolar traces from the electrode closest to the insulated myobundle (0 mm) exhibited a significantly larger depolarization reflection during insulated track activation (AP #1) compared to atrial wall activation (AP #3). However, unipolar EGM traces from more distant electrodes (3 mm and 6 mm) primarily show depolarization reflections occurring simultaneously with atrial wall activation (AP #3), indicating reduced sensitivity to the micro-anatomical reentrant path at greater distances.

### Unipolar mapping of micro-anatomic reentries

Fig 2 shows examples of MEM using unipolar EGMs at different electrode spacings (1, 3, 6, and 9 mm) and varying distances from the atrial wall (0.25 and 1 mm). When the unipolar mapping catheter maintained good contact with the atrial wall (separation = 0.25 mm), the micro-anatomical reentry track was identifiable across all inter-electrode spacings, provided that at least one electrode was within 1 mm of the reentrant track. The LAT instants of the micro-reentrant path (90–180 ms) appear as horizontal lines in the LAT map, coinciding with regions where the common track-to-wall EGM reflection ratio exceeded 1.

When the mapping catheter had poor contact with the atrial wall (contact distance = 1 mm), the micro-anatomical reentry path was only partially identifiable for inter-electrode spacings of 1 mm and 3 mm, although larger 6 mm and 9 mm spacings produced LAT maps resembling a centrifugal propagation or focal type pattern. Even when significant EGM signal contributions from the reentrant myobundle were present (indicated by a track-to-wall ratio >1 (green-red)), EGM mapping failed to accurately depict the reentrant path activation, instead displaying focal activation patterns.

### Bipolar mapping of micro-anatomic reentries

**Fig 3:**
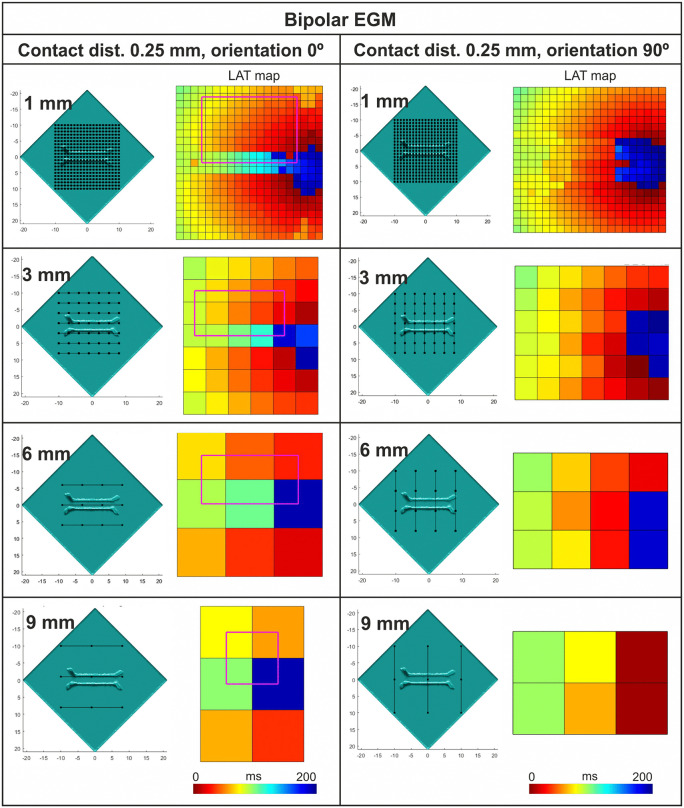
Bipolar mapping of micro-anatomical reentry with close EGM-to-Atria contact. Left and Right panels illustrate the schematic EGM positions relative to the reentrant structure and LAT maps, respectively, for each inter-electrode spacing (1, 3, 6, and 9 mm) and two bipolar orientations (0º and 90º, columns) with an EGM-to-atrial wall contact distance of 0.25 mm. Magenta rectangles on LAT maps represent the reentrant path identified, with the highest coverage of the cycle length.

**Fig 4:**
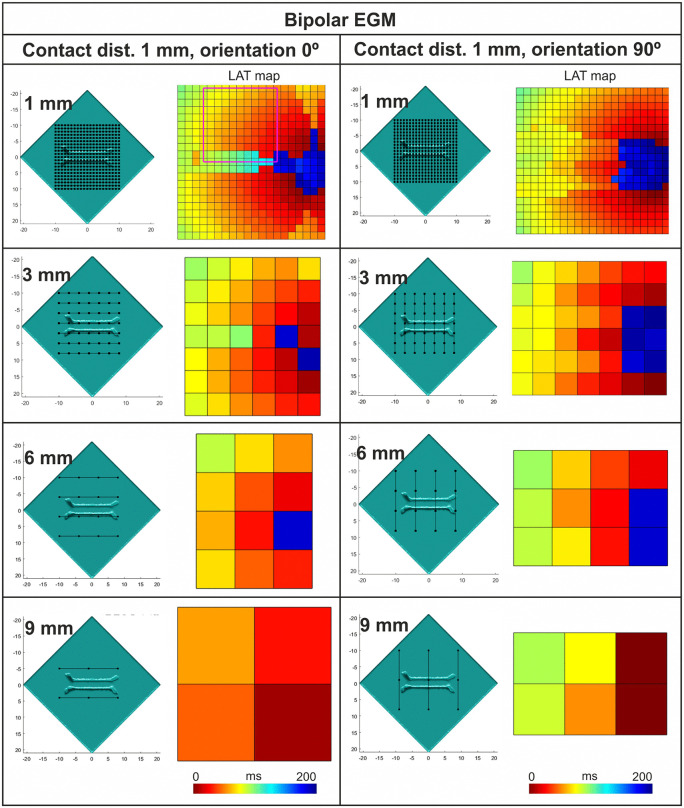
Bipolar mapping of micro-anatomical reentry with poor EGM-to-Atria contact. Left and Right panels illustrate the schematic EGM positions relative to the reentrant structure and LAT maps, respectively, for each inter-electrode spacing (1, 3, 6, and 9 mm) and two bipolar orientations (0º and 90º) with an EGM-to-atrial wall contact distance of 1 mm. Magenta rectangles on LAT maps represent the reentrant path identified, with the highest coverage of the cycle length.

The same in-silico experiment was performed using bipolar mapping catheters, as shown in Figs 3 and 4. Fig 3 illustrates the LAT map for bipolar catheter with good atrial wall contact (0.25 mm separation) under two different bipolar orientations: aligned with the micro-anatomical path (0º) and perpendicular to the micro-anatomical path (90º). The common reentry path was successfully mapped for all inter-electrode distances (1–9 mm), but only when the bipolar EGMs were aligned along with the micro-anatomical path. When the bipoles were perpendicular, the reentrant circuit was not seen, instead appearing as a focal activation pattern even at 1mm.

When the catheter had poor contact with the atrial wall (1 mm separation, Fig 4), the micro-anatomical path was identifiable only at the smallest inter-electrode spacing (1 mm) and only when the bipoles EGMs were aligned with the micro-anatomical path orientation.

### Omnipolar mapping of micro-anatomic reentries

**Fig 5:**
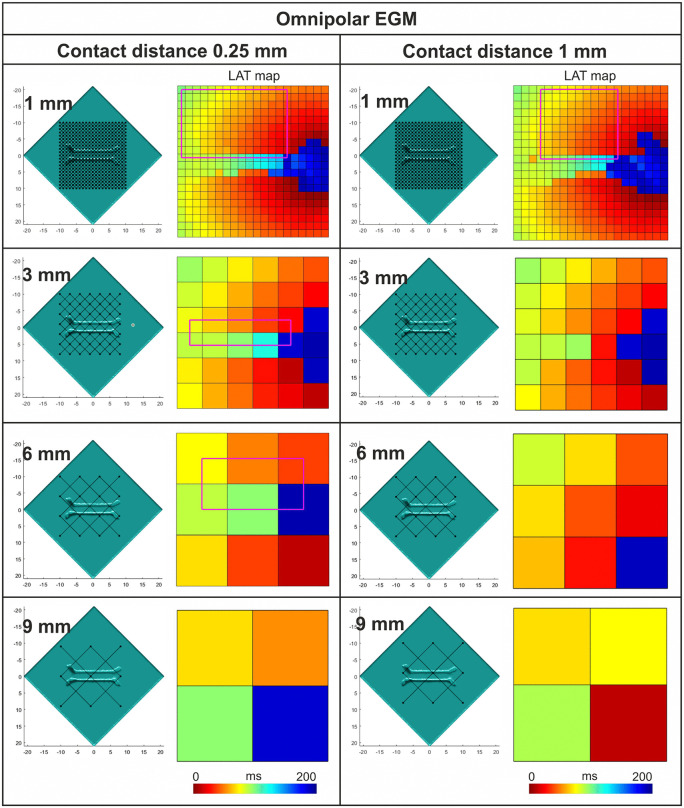
Omnipolar mapping of micro-anatomical reentry. Left and Right panels illustrate the schematic EGM positions relative to the reentrant structure and LAT maps, respectively, for each inter-electrode spacing (1, 3, 6, and 9 mm) and two EGM distances to the atrial wall (0.25 mm and 1 mm, columns). Magenta rectangles on LAT maps represent the reentrant path identified, with the highest coverage of the cycle length.

Fig 5 shows examples of MEM mapping using omnipolar traces, for different electrode spacing and distances to the atrial wall. When the catheter had good contact (0.25 mm), the micro-anatomical path was identifiable for all electrode spacing, although the lowest resolution (9 mm) missed part of the activation cycle. When the catheter was not in good contact with the atrial wall (1 mm), only the finest resolution (1 mm) was able to identify the micro-reentrant track, and the rest of the cases showed incomplete reentries (3 mm) or centrifugal propagations (6, 9 mm).

### Effects of mapping configuration on micro-reentrant path identification

**Fig 6:**
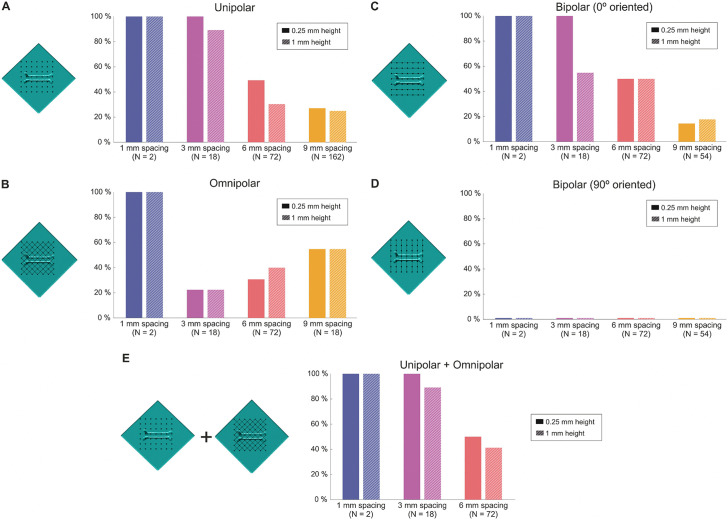
Percentage of cases identifying the micro-reentry based on EGMs catheter position and configuration. **A)** Unipolar mapping. **B)** Omnipolar mapping. **C)** Bipolar mapping oriented along with the micro-reentrant path. **D)** Bipolar mapping oriented 90º with respect to the micro-reentrant path. **E)** Combination of unipolar and omnipolar mapping.

To evaluate the effectiveness of different mapping approaches to automatically identify the micro-anatomical path, all possible EGM configurations were tested with 1 mm displacements from their original positions in the basal case (Fig 6). At 1 mm inter-electrode spacing, all MEM configurations successfully identified 100% of micro-reentrant tracks, except for misaligned bipolar mapping (Fig 6D). At 3–6 mm spacing, unipolar mapping identified the micro-reentrant path in 89% and 28%–47% of cases, respectively, with detection influenced by atrial wall contact. Omnipolar mapping at the same spacing (Fig 6B) had lower identification rates (22%–42%), similarly affected by atrial wall contact. Bipolar mapping demonstrated 47%–100% detection when aligned with the reentrant path and in good atrial wall contact (Fig 6C), but dropped to 0% when misaligned (Fig 6D).

At 9 mm spacing, identification ratios became less dependent on atrial wall contact (<10% variation). Omnipolar mapping had the highest detection rate (56%), followed by unipolar (26%–33%) and bipolar (22%–26% when aligned, 0% when misaligned at 90º).

Combining mapping modalities improved detection if at least one method successfully identified the micro-reentrant path. Unipolar combined with omnipolar mapping (Fig 6E) had the highest efficacy, achieving >90% detection for 3 mm spacing, ~ 50% for 6 mm, and wall contact produced changes in rate detection lower than 10%. MEM configurations with 9 mm spacing were excluded from the combined analysis due to limited cases (N = 9), though detection rates remained ~100%.

Among 82 cases analyzed with combined techniques, unipolar mapping detected the micro-reentrant path when other mapping techniques did not in 18 cases (22%), whereas omnipolar mapping improved detection in 10 cases (12%). Bipolar mapping had minimal impact, as only in 1 case (1%) bipolar mapping identified the micro-reentrant circuit, where neither unipolar nor omnipolar mapping identified it.

### Effect of substrate condition on micro-reentrant path identification

**Fig 7:**
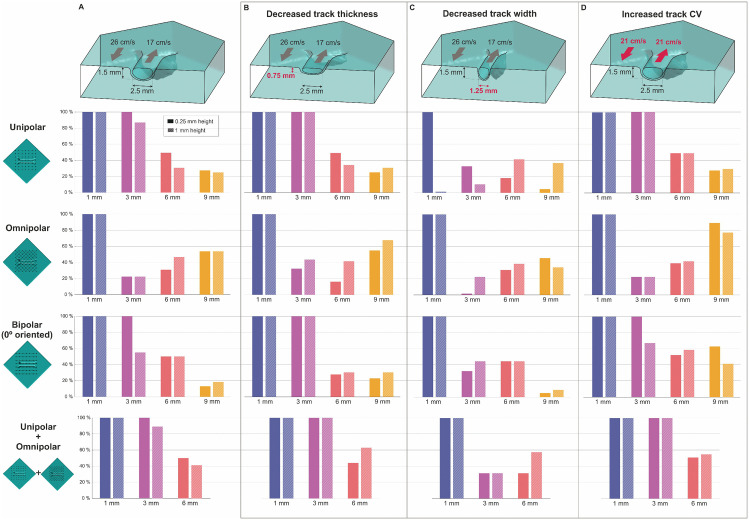
Micro-reentry detection under different substrate conditions. **A)** Baseline substrate. **B)** Micro-reentrant track with reduced thickness to 50% respect to baseline. **C)** Micro-reentrant track with reduced width to 50% respect to baseline. **D)** Micro-reentrant track with increased diffusion, so atrial wall and micro-reentrant track conduction velocity are similar.

In order to evaluate the effect of the atrial substrate on the detection of micro-reentries, in terms of the anatomical configuration of the micro-reentrant track and its electrical conduction conditions, simulations were performed on three other similar anatomical models with variations in the micro-reentrant anatomy and electrical diffusion (Fig 7).

In the simulation where the thickness of the micro-reentrant track was reduced to 50% (Fig 7B), the detection of the micro-reentry by the different mapping methods remained similar to the baseline case (Fig 7A), with variations of less than 20% in most cases. Detection via omnipolar mapping improved for spacings of 3 mm and 9 mm, but decreased for 6 mm, with similar overall results. In the bipolar case, the detection rate improved, especially for the 3-mm spacing with poor contact, which increased from 50% to 100%. Finally, for the combination of unipolar and omnipolar, the results were similar, with variations of around 10%.

When the width of the micro-reentrant path was reduced to 50% (Fig 7), the micro-reentrant identification outcomes showed significant differences compared to the baseline model. For unipolar mapping, the detection rate was significantly reduced, dropping in some cases from values close to 100% to less than 50%, across all spacings. In omnipolar mapping, the reductions in the detection rate were not as large, but considerable reductions (~30%) were also observed for the 3, 6, and 9 mm spacings, as well as in bipolar mapping. When considering unipolar and omnipolar mapping together, significant differences were observed for the 3-mm spacing, with detection rates dropping from 100% to 30%.

Finally, micro-reentry detection was evaluated in a simulation in which the conduction velocities of the micro-reentrant track and the atrial wall were aligned at approximately 21 cm/s (Fig 7D). Overall, this case showed a slight improvement over the baseline case. For unipolar mapping, the results of automatic detection were similar or slightly better (~10%). In omnipolar mapping, notable improvements were observed for the 9-mm spacing, where detection rates increased from 50% to 80%, similar to bipolar mapping. Combined unipolar and omnipolar detection also improved slightly (~10%) for 3- and 6-mm spacings.

### Mapping considerations

**Fig 8:**
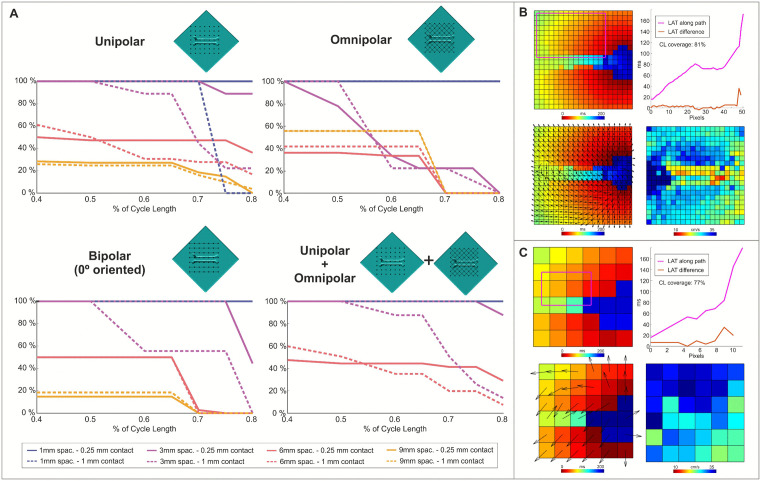
Mapping considerations for micro-reentry identification. **A)** Effect of threshold used for automatically considering a reentry, expressed as percentage of the cycle length, on the percentage of cases in which the micro-reentry was identifiable. Examples of micro-reentrant path identification on omnipolar mapping, for **(B)** 1 mm resolution and for **(C)** 3 mm resolution: top-left panel shows the omnipolar LAT map and the automatically-detected reentry (purple line); top-right panel shows the LAT sequence on the detected reentrant path (purple) and its first derivative (brown); bottom-left panel shows the propagation direction detected by omnipolar mapping (black arrows); bottom-right panel shows the conduction velocity map obtained with omnipolar mapping.

Various technical aspects related to mapping techniques can affect the detection of micro-reentrancy. In Fig 8A, we evaluate the effect on the detection rate of the threshold used by the automatic detection method, in terms of the percentage of cycle length present in the activation cycle required to effectively detect reentrancy. This threshold affects all mapping methods, since less strict values (lower percentage of the cycle length present in the reentry) result in higher detection rates, which stabilize around 50% of the cycle length. Stricter cycle length coverage values result in lower detection rates, which for many mapping scenarios approach 0% detection for >80% of the cycle, even for high-density mapping (1 mm spacing). This can be seen in Fig 8B and 8C, which show two examples of omnipolar mapping for spacings of 1 mm and 3 mm, respectively. The upper-left panel shows the rectangle with the greatest coverage of the detected cycle length (purple), and the upper-right panel shows the sequence of LATs detected in that panel, as well as their pixel-by-pixel variation (brown line). It can be observed that, particularly in the case of higher resolution (Fig 8B), there is a single step of ~30 ms corresponding to the LATs between 120 and 150 ms (light blue to dark blue on the LAT map), due to insufficient resolution in the reentrant path, resulting in a cycle length coverage of approximately 80%. A similar pattern can be observed in the case of lower resolution (8C), with a cycle length coverage of 77% in this case.

Other mapping modalities derived from omnipolar mapping provide additional information that can aid in the detection of this micro-reentry. This is the case with the propagation direction, which can be extracted from the omnipolar algorithm, as illustrated in the lower left panels of Fig 8B-C. It can be observed that the pixel-by-pixel propagation direction corresponds to that expected from the propagation sequence, although edge effects are observed at higher resolution (Fig 8B). The conduction velocity can also be extracted from this omnipolar algorithm (lower right panel in Fig 8B-C), which also allows to position the micro-reentrant path since it has a lower conduction velocity compared with the atrial wall. However, the lower-resolution omnipolar maps (Fig 8C) do not allow for a clear identification of the micro-reentrant path using this metric, due to the conduction velocity variations observed in the rest of the atrial wall.

## Discussion

This computational study systematically evaluated various MEM configurations, including inter-electrode distances, polarities, orientations, catheter-wall separation, and electrode positioning relative to the reentrant track, to determine the optimal conditions for identifying micro-reentrant circuits in AF mapping. Simulations were performed using a realistic anisotropic 3D model of a sub-endocardial fibrotically insulated myobundle sustaining AF [9], allowing for a detailed investigation of different mapping strategies and substrate conditions.

Our models demonstrated that micro-anatomic reentry exhibited distinct EGM patterns, particularly when electrodes were within 3 mm of the reentrant track and maintained close surface contact. In multiple-electrode MEM configurations, the reentrant track was consistently detected using dense unipolar MEMs (1–6 mm spacing). In bipolar catheter configurations, identification was only achieved when bipoles were aligned with the reentrant path, with detection rates comparable to unipolar configurations under these conditions. Omnipolar mapping provided no additional advantage over unipolar mapping at 1–6 mm spacing, but demonstrated improved identification rates at 9 mm spacing, suggesting its potential role in larger-spaced electrode configurations.

Regarding the substrate conditions, mapping was negatively affected by the micro-reentrant track width. Tracks with reduced width were harder to detect, although the combined mapping retained part of the detection even for micro-reentrant tracks considerably thinner than the electrode spacing (1.25 mm). Differences on the track thickness or conduction velocity did not negatively affect the micro-reentrant activity detection, and in some cases increased the detection ratio.

### Human AF maintenance by reentrant driver mechanisms

The presence of reentrant drivers in AF has been extensively studied using both epicardial and endocardial mapping approaches. Waldo’s group [21] conducted epicardial open-chest mapping studies in humans at 1.2-1.5 mm resolution, revealing focal waves characterized by wide distribution, non-repetitiveness, and initiation with an ‘R’ wave. While these features may suggest a focal origin, they can also be explained by transmural conduction with atrial surface breakthroughs (Figs 2-3, right panels). Simultaneous endocardial-epicardial MEM studies (2 mm resolution) during open-chest surgery revealed that asynchronous transmural wavefront propagation underlies the mechanism of most focal waves [22]. Simultaneous endo-epicardial MEM (3.5 – 5 mm resolution) of isolated canine atria during acetylcholine-induced AF found focal activation patterns simultaneously present on the endocardium and epicardium, separated by 1.5 cm [23]. These focal activation patterns could be attributed to small transmural reentry circuits using free-running bundles connecting the endocardium and epicardium. Evidences of AF maintenance due to atrial structures such as the pectinate bundles have also been reported using MEMs [16], including similar conditions (1.5 cm track, conduction velocity of 13–47 cm/s) to the ones simulated here. The relatively slow conduction velocities used in the micro-reentrant track were chosen to reproduce bundle-dependent slow conduction reported in prior experimental studies of atrial reentry. Similar conditions have been described for pectinate muscle-related intra-atrial reentry, with conduction velocities ranging from 13 to 47 cm/s [16], and in our *ex vivo* human optical mapping studies, where localized AF driver tracks of comparable dimensions showed conduction velocities around the reentrant circuit of 33 ± 14 cm/s [24] and micro-anatomic reentrant tracks measuring approximately 15.4 × 6.0 mm were directly identified in human atria [9].

Further evidence supporting intramural reentry has been obtained using near-infrared simultaneous sub-endocardial and sub-epicardial optical mapping studies, at sub-millimeter resolution, during persistent AF in both atria [7,9,10]. These studies have demonstrated the presence of subendocardial reentrant circuits within 3D fibrotic insulated myocardial bundles. Histologically validated 3D CE-MRI of optically-mapped AF driver regions suggests the 3D driver substrate or arrhythmogenic fibrotic hubs with anisotropic reentry pathways formed by the combination of intramural fibrotic strands, misaligned epi-endocardial myofibers (myofiber discontinuity), and varying atrial wall thickness [9,12]. These micro-anatomic intramural reentrant circuits are not confined to a single reentrant path but instead comprise multiple distinct pathways, including a common track for preferential conduction alongside alternative return pathways that complete the “figure-eight” reentrant circuit (Fig 1A) [25]. Ablation targeting the common micro-anatomic reentrant path has been observed to result in termination of AF, continuation of slower AF driven by a secondary reentrant driver or conversion of AF into macro-reentrant tachycardia. These findings suggest that intramural AF reentrant drivers may play a critical role in sustaining the fibrillatory process in human AF [2].

Our structural 3D model should be interpreted as a representative reduced geometry of a human AF driver region, not as an attempt to reproduce the full anatomical complexity of a specific RA or LA site. Human bi-atrial structure is regionally heterogeneous, with chamber-specific differences in wall thickness, wall-thickness variation, bundle architecture, fibrosis distribution, and transmural myofibre organization that may all affect extracellular electrograms. The right atrium can be regionally thicker and may contain more complex myofiber organization with bundles running in different directions, whereas the left atrial wall may show different thickness and fiber arrangement depending on the region studied. Because such structural differences can affect EGM amplitude, morphology, and spatial sampling, our model should be interpreted as a proof-of-principle framework intended to isolate the effects of track geometry and electrode spacing rather than to reproduce the full anatomical complexity of a specific atrial chamber. By focusing on the shared micro-anatomic reentrant track/common-track substrate identified in prior *ex vivo* human studies [7,9,10,12,13], the present model isolates the specific effects of catheter configuration on detectability while maintaining direct structural relevance to both RA and LA driver regions.

### Biophysical and technical considerations

We demonstrated that catheter configurations with inter-electrode distances greater than the micro-bundle width (3–6 mm) might be able to detect micro-reentries through EGM analysis. The identification of micro-reentries was severely affected by the contact of the catheter with the atrial wall, as previously described for the detection of functional reentries [26], as well as by the micro-reentrant track width. In that study, Bartolucci et al. observed that the detection of functional reentries was also dependent on electrode density, finding that MEM densities of 3–6 mm were able to identify these fibrillatory mechanisms.

In addition, we showed that the type of mapping also provided different accuracy in micro-reentrant path identification. Diameter, length, inter-electrode spacing, and height have been reported to cause progressive degradation in mapping quality [27–28]. Bipolar mapping in our study was severely influenced by alignment, as is well known. However, it has been found that the behavior of omnipolar electrodes, which in principle better rejects the far field [29], does not provide better results than unipolar configurations. This may be caused by the lower resolution of omnipolar maps, as one electrode is effectively lost compared with unipolar, and their reduced field of view compared to unipolar mapping [30]. Additionally, omnipolar electrodes are still orientation-sensitive, due to their bipolar nature, and therefore the far-field rejection of omnipolar electrodes may not compensate for their misalignment with the micro-bundle, as observed in our simulations (45° offset). Although bipolar and omnipolar electrograms are constructed from unipolar EGMs, and therefore constructed from the same baseline information, their processing, allowing for example the rejection of the far field, makes the activation maps different and therefore their reentry detection rate. Other information derived from omnipolar mapping, such as conduction velocity or conduction direction, could also aid in the detection of micro-reentries, although this mapping modality can be also limited by conduction velocity edge effects and the relative conduction velocity between tissue and the tracking system.

### Limitations

While this study provides valuable insights into optimal MEM configurations for mapping AF drivers, certain limitations must be acknowledged. Simulations may not fully capture the complexities of *in vivo* human AF, where factors such as region-specific myofibers heterogeneity and fibrosis distribution, and dynamic electrophysiological changes during AF could influence mapping accuracy. However, the impact of fiber direction and tissue types on the same specific computational model were already evaluated in previous studies [19,31]. The effect of the far field in unipolar signals, including ventricular activity, has not been considered in the analysis.

All simulations involved a micro-reentrant path, which allowed us to validate the MEM maps against an EGM-independent ground truth. However, the performance of this type of high-density mapping should also be evaluated in other scenarios without micro-reentries, such as endo-epicardial dissociation or fibrotically-provoked irregular propagation, in order to quantify the occurrence of false positives. Throughout the manuscript, a minimum resolution of 1 mm was considered, as it constitutes an idealized yet technically achievable scenario for clinical mapping and falls comfortably below commercially available resolutions. It should be noted, however, that sub-millimeter resolutions may offer further improvements in mapping accuracy. Electrode contact with the atrial wall was only evaluated for binary surrogates of good (0.25 mm) and bad (1 mm) contact, but further contact configurations should be evaluated. Moreover, other potential effects of the anatomical conformation should be investigated, such as the alignment of the electrode and myobundle, curved surfaces of electrode and/or atrial wall, and anatomical irregularities in the atrial wall.

EGM artifacts caused by discontinuous depolarization at the simulation edge (edge effects) can also affect the EGM shape, and therefore the detection of micro-reentries, in a virtual environment. However, for this study, this effect was evaluated under pacing conditions, and the edge effect artifact was found to be significantly smaller (<10%) than the actual depolarization amplitude. This study focused on a specific 3D cellular model of AF reentry, providing a cycle length equivalent to persistent AF, which may limit the generalizability of the findings to other AF driver subtypes. Furthermore, the study relied on simulated electrograms rather than patient-derived data, which may not fully reflect the variability encountered in clinical practice.

### Clinical implications

Optimal MEM configurations may help to improve the accuracy of mapping procedures, leading to more targeted and effective ablation therapies. Proximity of electrodes to the reentrant path, in terms of contact between the electrode and the atrial wall, is crucial for visualizing AF reentrant drivers, so this may imply changes to the current mapping protocols and devices to ensure the best contact [32]. Current high-density mapping catheters [2,33] may need to integrate unipolar and omnipolar mapping approaches during AF to enhance the detection of circuits sustaining AF.

Our findings advocate for a strategic rather than purely density-driven approach to AF mapping. Given that driver locations are patient-specific, initial wide-area panoramic mapping is necessary to identify regions potentially driving AF, such as with high dominant frequency or dispersed EGMs. Subsequent high-density interrogation of these areas should prioritize signal quality and catheter orientation, potentially utilizing unipolar and omnipolar integration to better detect localized reentry. Refinement may require iterative remapping with fine adjustments to catheter position and angle to account for local myofiber architecture. As micro-anatomic reentry cannot be visualized during sinus rhythm, this methodology is specifically intended for use during AF to improve the interpretation of suspected substrate. Consequently, clinical success depends not only on electrode density and contact but on a systematic workflow that transitions from broad regional identification to focused, multi-orientational remapping. Incorporating these insights into clinical practice could enhance perAF ablation success rates while potentially reducing recurrence and complications associated with AF ablation.

## Conclusions

This computational study simulated sustained AF episodes to determine the optimal MEM configurations for identifying the experimentally defined common track of micro-anatomical reentry sustaining human AF. Dense unipolar MEMs (1–3 mm spacing) reliably identified the reentrant track, while bipolar MEMs required bipoles to be aligned with the myobundle for accurate detection.

Omnipolar catheter configurations were equivalent to unipolar mapping for dense MEMs (3–6 mm spacing) but exhibited significantly better identification rates at larger spacings (9 mm). Mapping was hindered by narrow micro-reentrant tracks, though reentrant mapping still detected tracks much thinner than the electrode spacing. Combining unipolar and omnipolar mapping in dense MEM configurations (3–6 mm spacing) may be the most effective strategy for identifying micro-anatomical reentrant paths. This approach could enhance the success rates of perAF ablation procedures by enabling more precise localization of arrhythmogenic circuits.
