# Supplementary material for: Catheter configuration for mapping micro-anatomic reentries sustaining atrial fibrillation: A simulation study
Source: PLoS Comput Biol. 2026 Jul 24;22(7):e1014493. doi: 10.1371/journal.pcbi.1014493 (PMC13399530; doi:10.1371/journal.pcbi.1014493)
Supplement: Attachment — Submitted filename: 260504_Reviewer response.pdf [file pcbi.1014493.s002.pdf]

The authors sincerely appreciate the insightful comments and suggestions provided during this review, which have further improved the manuscript. The reviewers' questions are presented in bold, followed by our responses, and changes to the manuscript in italics.

Reviewer #1:

This paper computes electrograms over a cardiac muscular bridge. It examines how electrode spacing affects the ability to detect the structure. The authors examine the effect of spacing on several electrode types and conclude that a certain resolution is required for its detection. The situation modelled is very simple and does not capture the full physiological detail, but it may be sufficient for the point that the authors are trying to make. The conclusions reached by the authors are as expected. The paper is well organized and logical. The English needs improvement in a few places. Concerns about the paper are given below:

The authors need to describe how they processed signals for the omnipole and used them. The authors treat the LATs obtained from such as being based on bipoles, but they are derived from unipoles (DOI:10.1161/CIRCEP.116.004107) and used to construct the virtual bipoles. Thus, LATs as derived from omnipoles should never be worse than unipoles. Also, the authors are ignoring the additional information obtained from the omnipole such as CV and direction.

We appreciate the feedback and apologize for the lack of clarity in this area. We have improved the methodological description of the omnipolar EGM calculation (Material and methods, Sections *Electrogram calculation* and *Multi-electrode mapping*):

> *Bipolar EGMs were calculated as the difference between unipolar EGM traces at different spacing, calculated in the same direction (0º) and perpendicular (90º) to the sub-endocardial micro-reentrant path. Omnipolar EGM traces were calculated using groups (cliques) of 4 electrodes in 2 $\times$ 2 arrangements, in which diagonal bipoles were used to construct omnipolar EGM traces [20]. Omnipolar EGMs were computed as the orthogonal transformation that maximizes the amplitude of the activation. This omnipolar disposition provides equivalent resolution to unipolar and bipolar EGM maps, and it has been defended as more robust for Local Activation Time (LAT) estimation [20].*
>
> *[…]*
>
> *On unipolar EGM maps, LAT was calculated by identifying the activation as the instant with maximal negative derivative of voltage over time. On bipolar and omnipolar EGM traces, LAT was identified as the instant with maximal absolute amplitude. Omnipolar calculation was used also to identify conduction velocity and propagation direction.*

Following the reviewer's recommendations, we have also included a new figure (Fig. 8), in which we evaluate the additional information provided by the omnipolar mapping (Results, Section *Mapping considerations*):

> *Other mapping modalities derived from omnipolar mapping provide additional information that can aid in the detection of this micro-reentry. This is the case with the propagation direction, which can be extracted from the omnipolar algorithm, as illustrated in the lower left panels of Figure 8B-C. It can be observed that the pixel-by-pixel propagation direction corresponds to that expected from the propagation sequence, although edge effects are observed at higher resolution (Figure 8B). The conduction velocity can also be extracted from this omnipolar algorithm (lower right panel in Figure 8B-C), which also allows to position the micro-reentrant path since it has a lower conduction velocity compared with the atrial wall. However, the lower-resolution omnipolar maps (Figure 8C) do not allow for a clear identification of the micro-reentrant path using this metric, due to the conduction velocity variations observed in the rest of the atrial wall.*

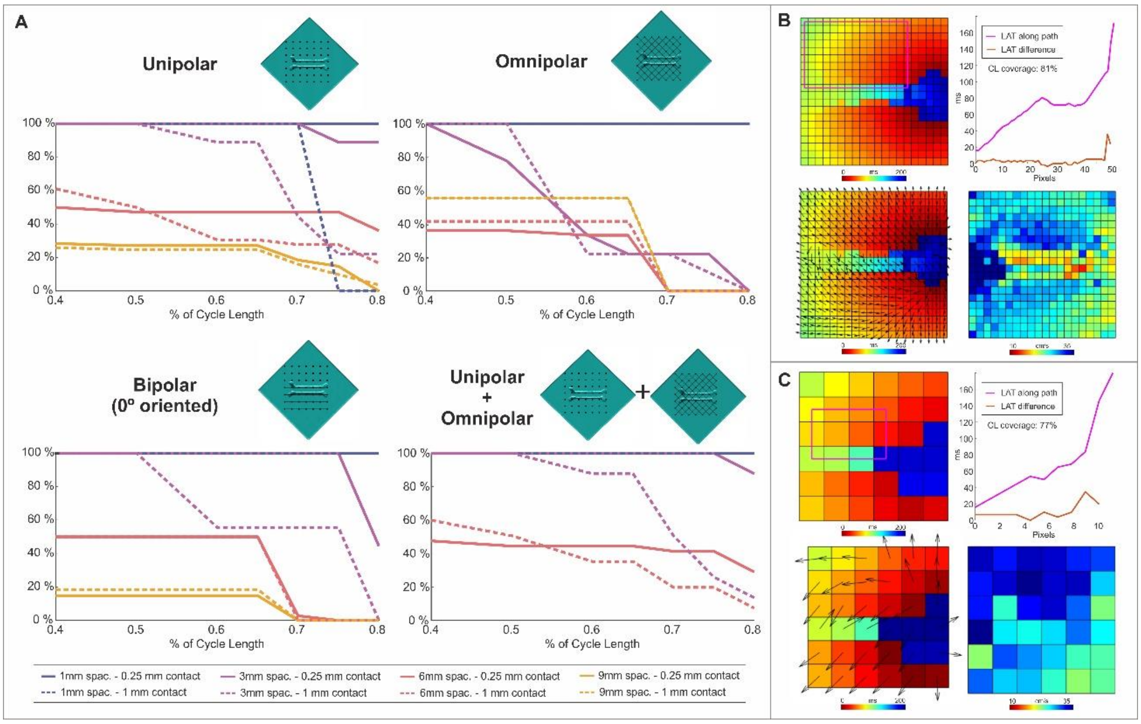

Figure 8. Mapping considerations for micro-reentry identification. A) Effect of threshold used for automatically considering a reentry, expressed as percentage of the cycle length, on the percentage of cases in which the micro-reentry was identifiable. Examples of micro-reentrant path identification on omnipolar mapping, for (B) 1mm resolution and for (C) 3mm resolution: top-left panel shows the omnipolar LAT map and the automatically-detected reentry (purple line); top-right panel shows the LAT sequence on the detected reentrant path (purple) and its first derivative (brown); bottom-left panel shows the propagation direction detected by omnipolar mapping (black arrows); bottom-right panel shows the conduction velocity map obtained with omnipolar mapping.

This has been also addressed in the discussion (Section *Biophysical and technical considerations*):

> *Although bipolar and omnipolar electrograms are constructed from unipolar EGMs, and therefore constructed from the same baseline information, their processing, allowing for example the rejection of the far field, makes the activation maps different and therefore their reentry detection rate. Other information derived from omnipolar mapping, such as conduction velocity or conduction direction, could also aid in the detection of micro-reentries, although this mapping modality can be also limited by conduction velocity edge effects and the relative conduction velocity between tissue and the tracking system.*

Some of the parameters which influence the EGM seem arbitrary. For example, the bridge conduction velocity is set lower than the wall, which will lower its EGM amplitude. Also, the tissue thickness will affect the ratio of the signals. The authors conclusions about electrode spacing distance also seem dependent on the width of the bridge used. A thinner bridge would require finer sampling.

We appreciate the feedback provided, as it has allowed us to significantly improve the manuscript's content. Following the reviewer's suggestion, we have included simulations covering other anatomical configurations of the micro-reentrant track: smaller thickness, smaller depth, and higher conduction velocity. This has been included in a new figure (Fig. 7)

and the following text has been added to a new Results section *Effect of substrate condition on micro-reentrant path identification*:

In order to evaluate the effect of the atrial substrate on the detection of micro-reentries, in terms of the anatomical configuration of the micro-reentrant track and its electrical conduction conditions, simulations were performed on three other similar anatomical models with variations in the micro-reentrant anatomy and electrical diffusion (Figure 7).

In the simulation where the thickness of the micro-reentrant track was reduced to 50% (Figure 7B), the detection of the micro-reentry by the different mapping methods remained similar to the baseline case (Figure 7A), with variations of less than 20% in most cases. Detection via omnipolar mapping improved for spacings of 3 mm and 9 mm, but decreased for 6 mm, with similar overall results. In the bipolar case, the detection rate improved, especially for the 3-mm spacing with poor contact, which increased from 50% to 100%. Finally, for the combination of unipolar and omnipolar, the results were similar, with variations of around 10%.

When the width of the micro-reentrant path was reduced to 50% (Figure 7), the micro-reentrant identification outcomes showed significant differences compared to the baseline model. For unipolar mapping, the detection rate was significantly reduced, dropping in some cases from values close to 100% to less than 50%, across all spacings. In omnipolar mapping, the reductions in the detection rate were not as large, but considerable reductions (~30%) were also observed for the 3, 6, and 9 mm spacings, as well as in bipolar mapping. When considering unipolar and omnipolar mapping together, significant differences were observed for the 3-mm spacing, with detection rates dropping from 100% to 30%.

Finally, micro-reentry detection was evaluated in a simulation in which the conduction velocities of the micro-reentrant track and the atrial wall were aligned at approximately 21 cm/s (Figure 7D). Overall, this case showed a slight improvement over the baseline case. For unipolar mapping, the results of automatic detection were similar or slightly better (~10%). In omnipolar mapping, notable improvements were observed for the 9-mm spacing, where detection rates increased from 50% to 80%, similar to bipolar mapping. Combined unipolar and omnipolar detection also improved slightly (~10%) for 3- and 6-mm spacings.

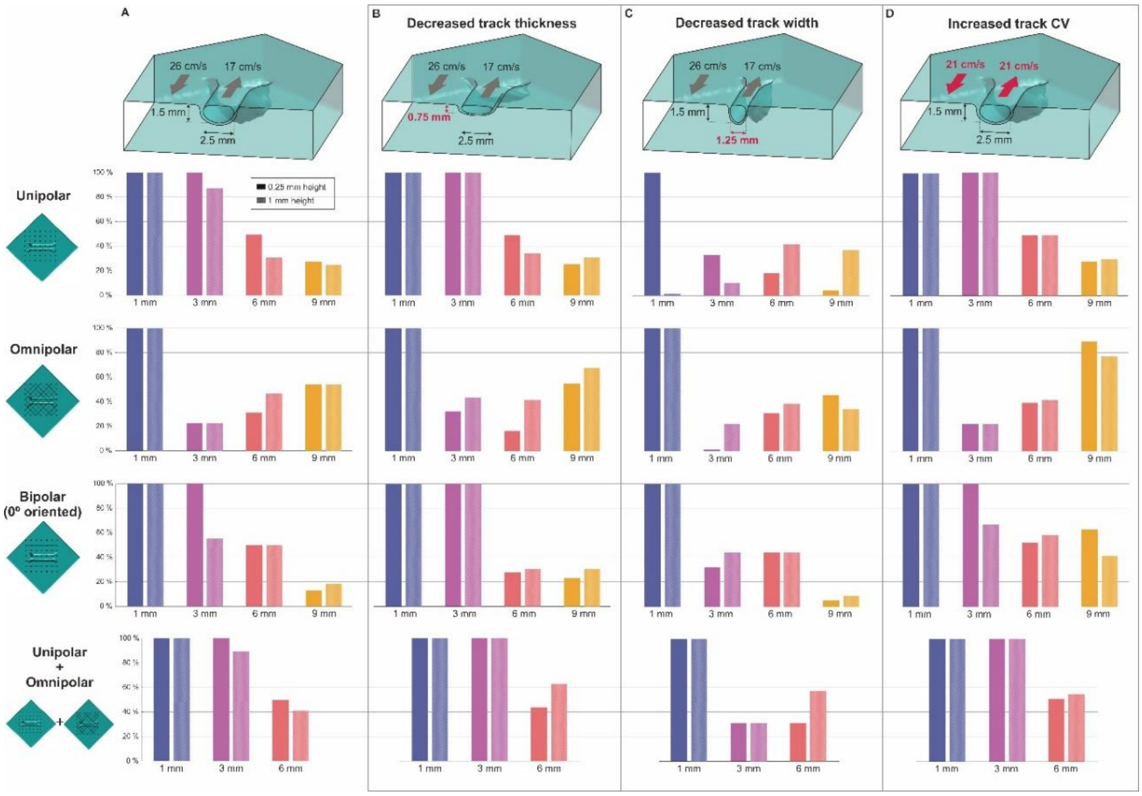

Figure 7. Micro-reentry detection under different substrate conditions. A) Baseline substrate. B) Micro-reentrant track with reduced thickness to 50% respect to baseline. C) Micro-reentrant track with reduced width to 50% respect to baseline. D) Micro-reentrant track with increased diffusion, so atrial wall and micro-reentrant track conduction velocity are similar.

This was also commented on the discussion:

> *Regarding the substrate conditions, mapping was negatively affected by the micro-reentrant track width. Tracks with reduced width were harder to detect, although the combined mapping retained part of the detection even for micro-reentrant tracks considerably thinner than the electrode spacing (1.25mm). Differences on the track thickness or conduction velocity did not negatively affect the micro-reentrant activity detection, and in some cases increased the detection ratio.*

The authors do not specify a location for the tissue they are simulating other than atrial. This is important due to the difference in structure between the atria. The RA layer tends to be thinner with all fibers in the same direction, and muscle bundles laid on top of that. The LA is thicker with two distinct layers of differently oriented fibres, which will affect EGMs.

We thank the reviewer for this important comment. We agree that atrial location and structural context should be stated more clearly, because RA and LA differ in wall thickness, wall-thickness variation, bundle architecture, and transmural myofibre organization, all of which may influence extracellular electrograms.

In the revised manuscript, we now clarify that the simulated tissue was not intended to reproduce one specific anatomical site of either atrium. Rather, it was constructed as a simplified but representative human atrial driver-region geometry, based on the characteristic micro-anatomic reentrant track/common-track configuration revealed by prior human ex vivo structural-functional studies. Thus, the aim of the model was not to reproduce the full chamber-

specific anatomy of the RA or LA, but to capture the essential 3D structural configuration relevant to both RA and LA driver regions and to isolate how electrode spacing, orientation, and wall contact affect its detectability on MEM recordings. We addressed this point in the Material and Methods section (Section *Biophysical simulation of a micro-anatomical reentry*):

> *Based on prior ex vivo human studies of atrial driver regions [7, 9, 11-13], we constructed a simplified but representative human atrial driver-region geometry rather than a full chamber-specific reconstruction of either the right atrium (RA) or left atrium (LA). The model was designed to capture the essential 3D structural configuration shared by human atrial driver substrates, including a sub-endocardial, laterally-insulated myobundle reproducing the essential common micro-anatomic reentrant track configuration described in prior structural-functional studies. This reduced geometry was selected to preserve the key substrate features relevant to both RA and LA driver regions while allowing controlled evaluation of how electrode spacing, orientation, and wall contact influence MEM-based detection of the reentrant track. Based on the 3D structure of AF driver reentrant track revealed by ex vivo human heart 3D imaging [9], we constructed a 3D anatomical model consisting of a wedge of atrial wall tissue (30 × 30 × 4 mm) with a sub-endocardial, laterally-insulated myobundle (15 × 2.5 × 1.5 mm) (Figure 1), embedded in the atrial wall tissue.*

We also addressed this point in the discussion's section (Section *Human AF maintenance by reentrant driver mechanisms*):

> *Our structural 3D model should be interpreted as a representative reduced geometry of a human AF driver region, not as an attempt to reproduce the full anatomical complexity of a specific RA or LA site. Human bi-atrial structure is regionally heterogeneous, with chamber-specific differences in wall thickness, wall-thickness variation, bundle architecture, fibrosis distribution, and transmural myofibre organization that may all affect extracellular electrograms. The right atrium can be regionally thicker and may contain more complex myofiber organization with bundles running in different directions, whereas the left atrial wall may show different thickness and fiber arrangement depending on the region studied. Because such structural differences can affect EGM amplitude, morphology, and spatial sampling, our model should be interpreted as a proof-of-principle framework intended to isolate the effects of track geometry and electrode spacing rather than to reproduce the full anatomical complexity of a specific atrial chamber. By focusing on the shared micro-anatomic reentrant track/common-track substrate identified in prior ex vivo human studies [7, 9-10, 12-13], the present model isolates the specific effects of catheter configuration on detectability while maintaining direct structural relevance to both RA and LA driver regions.*

How do the authors get 336k nodes? With 0.267 mm edge lengths, I calculate something closer to 191k nodes.

We understand that the estimate of 191k nodes is based on the assumption of a regular hexahedral mesh, with cubes of the same dimensions on all axes (0.26 × 0.26 × 0.26 mm), which is not the case for this model. In our case, we use a tetrahedral mesh, with distances between nodes that adapt to the irregularity of some parts of the model increasing the node density, so the total number of nodes is significantly higher. The geometric model used in this manuscript is published and available as open Access, as stated on the Data Availability section, at https://doi.org/10.5281/zenodo.14989415

Was the myobundle placed on top of the slab or within it? Were the electrodes in the same plane, or were electrodes over the myobundle displaced a further 1.5 mm in the normal direction.

We apologize for the lack of clarity on this point. We have provided a more detailed description of this aspect in the Materials and Methods section (Sections *Biophysical simulation of a micro-anatomical reentry* and *Electrogram calculation*), and the new Figure 7, included 3 comments above, shows a cross-section where this aspect is clearly visible.

> *[…] we constructed a 3D anatomical model consisting of a wedge of atrial wall tissue (30 × 30 × 4 mm) with a sub-endocardial, laterally-insulated myobundle (15 × 2.5 × 1.5 mm) (Figure 1), embedded in the atrial wall tissue.*
>
> …
>
> *Simulated unipolar EGMs were calculated using cylindrical electrodes (0.5 mm radius, 1 mm length, similar to grid and basket-type commercial catheters) positioned on a 2D plane above the endocardial surface, using two different distances in the normal direction (0.25, 1 mm) between these electrodes and endocardial wall where the myobundle is embedded.*

What are the authors trying to say in lines 18-22. Please rephrase with complete sentences. It is not clear.

We apologize for the lack of clarity. However, the reference to lines 18-22 is ambiguous, as each page has its own line numbering. Based on this description, we have identified two potential sections that may be unclear (Materials and Methods, Section *Biophysical simulation of a micro-anatomical reentry* and Discussion, Section *Human AF maintenance by reentrant driver mechanisms*), which we have proceeded to clarify:

> *Anisotropy of the cardiac tissue was applied varying the longitudinal and transversal diffusion (1:1.36 ratio in conduction velocity) [17-18] respect to the fiber direction, which was constant and aligned with the laterally-insulated myobundle orientation. Diffusion tensor was calculated with its maximal component D_{long} aligned with the fiber direction and the minimal D_{trans}, perpendicular to the fiber direction.*
>
> …
>
> *Ablation targeting the common micro-anatomic reentrant path has been observed to result in termination of AF, continuation of slower AF driven by a secondary reentrant driver or conversion of AF into macro-reentrant tachycardia. These findings suggest that intramural AF reentrant drivers may play a critical role in sustaining the fibrillatory process in human AF [2].*

A cycle length of 174 ms seems too long, as using the given conduction velocities (17 and 26 cm/s), the time for a reentry would be about 145 ms. Do the authors have references for these particular velocities? They are quite low.

We thank the reviewer for this important comment. The reviewer is correct that, based on the reported conduction velocities alone, a somewhat shorter cycle length might be expected. However, the effective reentrant path in the simulation was longer than the physical length of the myobundle because propagation was not uniform in some parts of the circuit. This has been better explained in Results, Section *Electrogram Traces on Micro-Reentrant Track:*

Partial conduction block and source–sink mismatch at the exit of the micro-reentrant track produced discontinuous propagation, increasing the effective reentrant path length to ~36 mm, slightly longer than twice the physical myobundle length of 15 mm.

Regarding the conduction velocities, these values were selected to reproduce slow conduction conditions reported for atrial bundle-dependent reentry and human micro-anatomic AF driver substrates, rather than uniform conduction in ordinary atrial myocardium. In support of this, (Wu et al., Circulation Research, 1998) reported pectinate muscle bundle–dependent intra-atrial reentry with conduction velocities ranging from 13 to 47 cm/s. In addition, our ex vivo human optical mapping studies demonstrated comparable dimensions and conduction properties of localized AF driver tracks. (Hansen et al., European Heart Journal, 2015) identified human intramural micro-anatomic reentrant tracks measuring approximately 15.4 ± 2.2 × 6.0 ± 2.3 mm with 2.9 ± 0.9 mm depth. More directly, (Li et al., Circulation, 2016) measured the conduction velocity around a human AF reentrant driver region in ex vivo optical mapping and reported an average conduction velocity of 33 ± 14 cm/s, as shown in the online-only Data Supplement Figure IV. Thus, the conduction velocities used in the present simulations fall within the experimentally observed range for human atrial micro-reentrant driver regions. This was discussed in the manuscript in Discussion, Section *Human AF maintenance by reentrant driver mechanisms*:

Evidences of AF maintenance due to atrial structures such as the pectinate bundles have also been reported using MEMs [16], including similar conditions (1.5 cm track, conduction velocity of 13 to 47 cm/s) to the ones simulated here. The relatively slow conduction velocities used in the micro-reentrant track were chosen to reproduce bundle-dependent slow conduction reported in prior experimental studies of atrial reentry. Similar conditions have been described for pectinate muscle-related intra-atrial reentry, with conduction velocities ranging from 13 to 47 cm/s [16], and in our ex vivo human optical mapping studies, where localized AF driver tracks of comparable dimensions showed conduction velocities around the reentrant circuit of 33 ± 14 cm/s [24] and micro-anatomic reentrant tracks measuring approximately 15.4 × 6.0 mm were directly identified in human atria [9].

Did displacing the 1 mm grid by 1 mm do anything? Would it not have been better to displace the grids by half of their sampling resolution?

In the manuscript, we have considered a minimum resolution of 1 mm, as it represents an ideal, technically feasible scenario for clinical mapping but at the same time is sufficiently far below commercially available resolutions. The analysis of the endocardial maps at 1 mm resolution did not involve any displacement, and a single map was evaluated, as can be observed on new Table 1. This was commented on the discussion (Section *Limitations*):

Throughout the manuscript, a minimum resolution of 1 mm was considered, as it constitutes an idealized yet technically achievable scenario for clinical mapping and falls comfortably below commercially available resolutions. It should be noted, however, that sub-millimeter resolutions may offer further improvements in mapping accuracy.

Are edge effects seen in the EGMs, as the excitation/repolarization wavefronts hit the edges?

We appreciate the suggestion, as effects on the EGM caused by the discontinuity at the simulation edges can affect the micro-reentry detection in our scenario. This is something we took into account when designing the experiment, and which we evaluated before proceeding with the rest of the study. To do this, we evaluated pacing simulations from the exit point of the micro-reentrant path, at a slower pacing rate (450 ms), that allowed us to visualize this effect in the EGM (purple point), as can be seen in the following figure. When the depolarization front

reached the simulation edges, there were slight deflections on the EGM, but their amplitude was lower than the pacing artifact (450 ms).

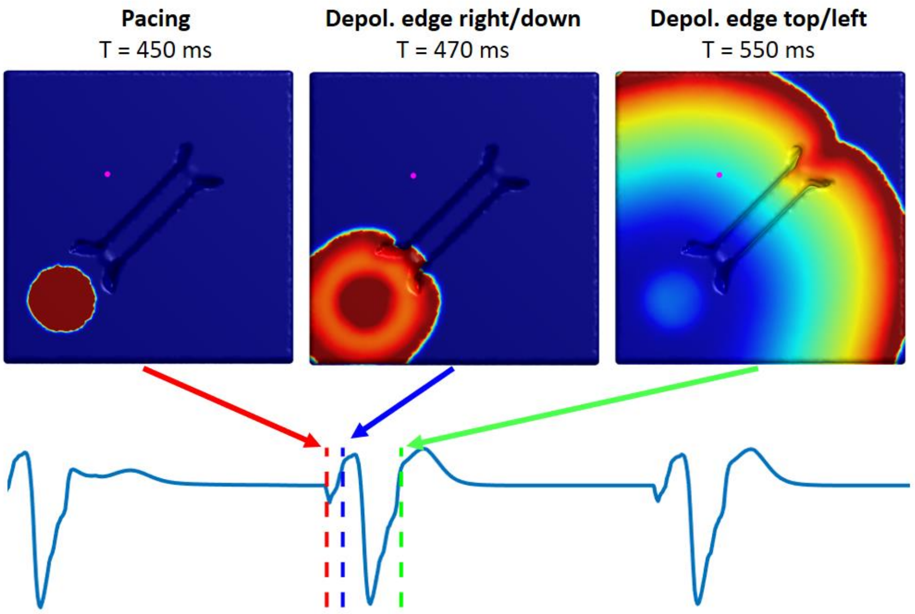

We included a comment in the limitations section about this point:

> *EGM artifacts caused by discontinuous depolarization at the simulation edge (edge effects) can also affect the EGM shape, and therefore the detection of micro-reentries, in a virtual environment. However, for this study, this effect was evaluated under pacing conditions, and the edge effect artifact was found to significantly smaller (<10%) than the actual depolarization amplitude.*

The authors could also comment on previous research looking at optimal electrode spacing.

Following the reviewer suggestion, we discussed previous findings in the discussion (Section *Biophysical and technical considerations*):

> *Diameter, length, inter-electrode spacing, and height have been reported to cause progressive degradation in mapping quality [27-28]. Bipolar mapping in our study was severely influenced by alignment, as is well known. However, it has been found that the behavior of omnipolar electrodes, which in principle better rejects the far field [29], does not provide better results than unipolar configurations. This may be caused by the lower resolution of omnipolar maps, as one electrode is lost respect to unipolar, and their reduced field of view compared to unipolar mapping [30].*

Reviewer #2:

PEER REVIEW: PCOMPBIOL-D-25-01546

Title: Catheter Configuration for Mapping Micro-Anatomic Reentries sustaining Atrial Fibrillation: a simulation study

SUMMARY

This computational study evaluates multi-electrode mapping (MEM) configurations for detecting micro-anatomic reentrant circuits that purportedly sustain atrial fibrillation. While the simulation methodology is straightforward and the technical analysis of electrode configurations is present, the paper has significant limitations regarding the clinical relevance of the substrate being modeled, selective citation of supporting evidence, and subjective outcome measures.

MAJOR CONCERNS

1. Limited Independent Evidence for Micro-Reentrant Driver Hypothesis

The paper's premise rests heavily on the concept that AF is maintained by intramural micro-anatomic reentrant circuits within fibrotically-insulated myobundles. However, the supporting evidence comes predominantly from the authors' own optical mapping studies [refs 6, 8, 9, 19].

Indeed, when evidence is considered in aggregate in conjunction with contemporary clinical practice as evidenced by the HRS/EHRA/APHRS current consensus statement there is a paucity of randomised trial evidence supporting the use of any form of driver based ablation; none of the lead authors of the contemporary clinical consensus publicly supports the use of driver based ablation.

This is a fundamental and serious limitation of the manuscript.

We thank the reviewer for this comment. Micro-reentrant pathways are proposed here as one possible mechanism contributing to AF perpetuation, complementary to other established mechanisms such as multiple wavelets, endo-epicardial dissociation, and functional reentry, among others. We have chosen to focus specifically on micro-reentry because its detection is constrained by current mapping technology, making it particularly amenable to *in silico* investigation. The introduction has been revised accordingly and now reads as follows:

> *Primary theories regarding the maintenance mechanisms of persistent AF (perAF) revolve around either multi-wavelets propagating across the atria or localized AF drivers, electrical phenomena critical to AF perpetuation [2]. Posteriorly, AF has been defended to be sustained by drivers characterized by rapid reentrant electrical activity that spreads throughout the atria and can be temporally stable or unstable [3]. Targeted ablation of AF drivers reduces the ability to sustain AF and has shown promise in improving treatment efficacy and long-term outcomes compared to conventional pulmonary vein isolation [4-5]. However, guided ablation strategies going beyond pulmonary vein isolation currently lack sufficient scientific evidence to be recommended in clinical practice [6]. Accurately identifying patient-specific AF drivers poses a challenge due to the inadequate visualization of potential intramural reentrant drivers using surface-only multi-electrode mapping (MEM) [7]. This limitation has resulted in conflicting clinical mapping results and outcomes in AF driver ablation trials [2, 8].*

2. Selective Presentation of Driver Ablation Efficacy

The introduction and discussion selectively cite studies supporting driver-targeted ablation [refs 4, 5] while omitting contradictory evidence. In practice, the interest of the clinical community in driver based ablation has declined.

We refer the reviewer to the answer to the previous comment.

3. Subjective Endpoint for Circuit Identification (Critical Limitation)

The study's primary outcome—"identification" of the micro-reentrant circuit—is defined as "reentrant LAT path in ≥4 electrodes representing >75% of the activation cycle" (page 7). This represents a subjective and arbitrary visual interpretation rather than an objective metric.

Major concerns:

• No quantitative validation against ground truth activation patterns

• No inter-observer reliability testing

• The 75% threshold appears arbitrary and unjustified

• The authors themselves determine which maps show "reentry"—potential for confirmation bias

We thank the reviewer for the comments provided. Following the reviewer's recommendation, we have included automatic reentry pattern detection for all analyses in the manuscript, as described in the Materials and Methods section (Section *Multi-electrode mapping*):

> *LAT maps with different electrode configurations were used to evaluate the micro-reentry presence. They were automatically evaluated by identifying rectangles along the LAT map with potential reentrant LAT paths, defined in ≥4 contiguous electrodes. An intensive search for all the rectangles in the LAT was carried out, and reentrant LAT paths were considered when the LAT values of the rectangular path presented a quasi-monotonic increasing/decreasing pattern, starting from the minimal LAT, with deviations < 3ms from monotonicity. The percentage of the reentrant sequence covering the cycle was calculated as the cycle length minus the highest increment between consecutive electrodes, in %. Micro-reentrant paths were identified in LAT maps when rectangular, reentrant paths covered >65% of the cycle length, and the rectangle with the highest coverage was selected (Figure 2). Maps with global LAT spanning <50% of the cycle length were not evaluated for reentrant paths.*

All figures on the manuscript now contain the automatically-identified reentrant path, as in the following example:

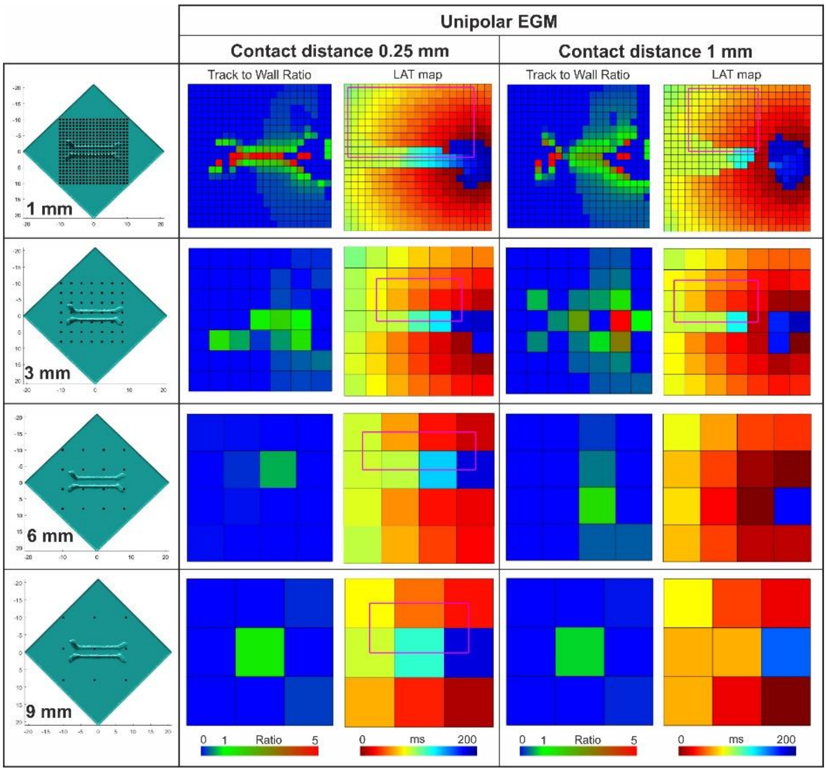

Figure 2. Unipolar EGM mapping of micro-anatomical reentry. Left panels illustrate the schematic EGM positions relative to the reentrant structure for each inter-electrode spacing (1, 3, 6, and 9 mm). Middle and right panels display the recorded reentry track-to-wall ratio and LAT maps, respectively, for each inter-electrode spacing (1, 3, 6, and 9 mm) and two EGM distances to the atrial wall (0.25 mm and 1 mm). Magenta rectangles on LAT maps represent the reentrant path identified, with the highest coverage of the cycle length.

In addition, we have also included a sensitivity study of the parameter used to automatically detect reentry, shown in a new Figure 8, and described in Results section (Section *Mapping considerations*):

Various technical aspects related to mapping techniques can affect the detection of micro-reentrancy. In Figure 8A, we evaluate the effect on the detection rate of the threshold used by the automatic detection method, in terms of the percentage of cycle length present in the activation cycle required to effectively detect reentrancy. This threshold affects all mapping methods, since less strict values (lower percentage of the cycle length present in the reentry) result in higher detection rates, which stabilize around 50% of the cycle length. Stricter cycle length coverage values result in lower detection rates, which for many mapping scenarios approach 0% detection for >80% of the cycle, even for high-density mapping (1mm spacing). This can be seen in Figures 8B and 8C, which show two examples of omnipolar mapping for spacings of 1 mm and 3 mm, respectively. The upper-left panel shows the rectangle with the greatest coverage of the detected cycle length (purple), and the upper-right panel shows the sequence of LATs detected in that panel, as well as their pixel-by-pixel variation (brown line). It can be observed that, particularly in the case of higher resolution (Figure 8B), there is a single step of ~30 ms corresponding to the LATs between 120 and 150 ms (light blue to dark blue on the LAT map), due to insufficient resolution in the reentrant path, resulting in a cycle length coverage of approximately 80%. A similar pattern can be observed in the case of lower resolution (8C), with a cycle length coverage of 77% in this case.

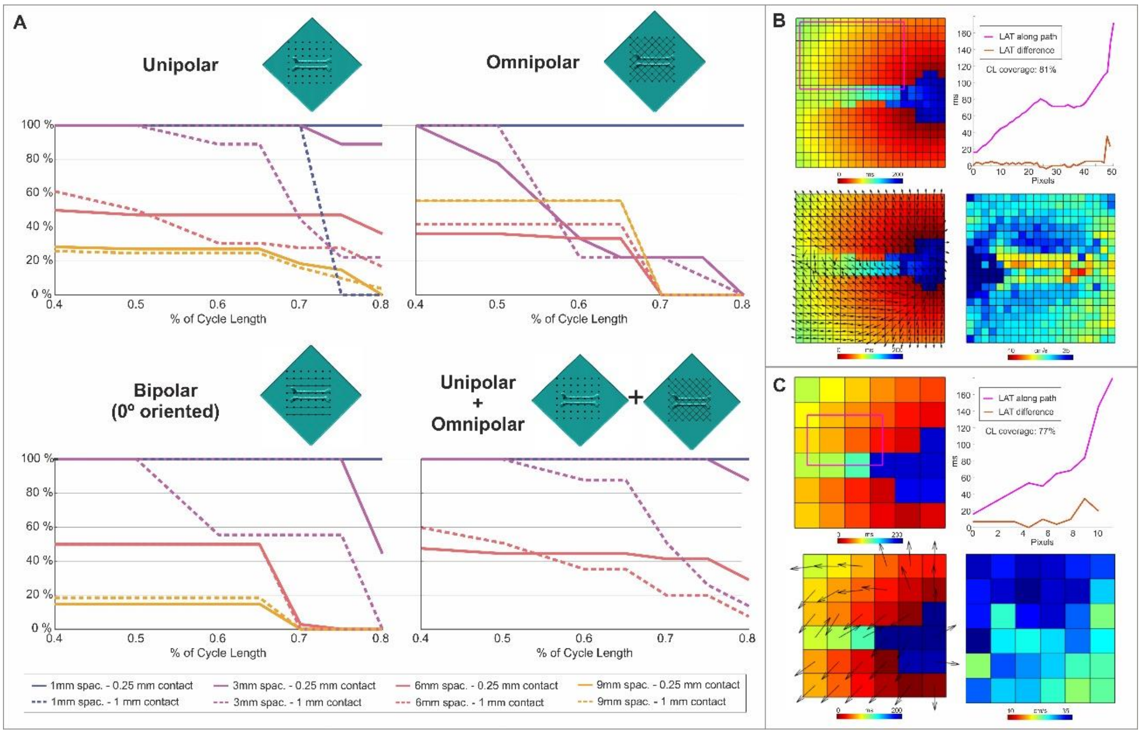

Figure 8. Mapping considerations for micro-reentry identification. A) Effect of threshold used for automatically considering a reentry, expressed as percentage of the cycle length, on the percentage of cases in which the micro-reentry was identifiable. Examples of micro-reentrant path identification on omnipolar mapping, for 1mm resolution (B) and for 3mm resolution (C): top-left panel shows the omnipolar LAT map and the automatically-detected reentry (purple line); top-right panel shows the LAT sequence on the detected reentrant path (purple) and its first derivative (brown); bottom-left panel shows the propagation direction detected by omnipolar mapping (black arrows); bottom-right panel shows the conduction velocity map obtained with omnipolar mapping.

Regarding the quantitative validation against ground truth activation patterns, all simulations were sustained by a reentry involving the micro-rentrant path, so the independent ground truth is inherent to the simulation protocol. We included a comment about this point in the limitation section:

> *All simulations involved a micro-reentrant path, which allowed us to validate the MEM maps against an EGM-independent ground truth. However, the performance of this type of high-density mapping should also be evaluated in other scenarios without micro-reentries, such as endo-epicardial dissociation or fibrotically-provoked irregular propagation, in order to quantify the occurrence of false positives.*

4. Relationship to Topological Principles

The simulated reentrant circuits appear to follow topological balance principles described by Vandersickel et al. and others,2-5: The application of these principles provides a more meaningful pathway to achieve clinical relevance.

We thank the reviewer for the suggestion, and we discussed this point in the discussion section (Section *Human AF maintenance by reentrant driver mechanisms*):

These micro-anatomic intramural reentrant circuits are not confined to a single reentrant path but instead comprise multiple distinct pathways, including a common track for preferential conduction alongside alternative return pathways that complete the "figure-eight" reentrant circuit (Figure 1A) [25].

5. Unclear Clinical Significance

The clinical translation pathway remains vague:

• Electrode spacing: Current HD catheters (HD grid Advisor, Rhythmia) use 2.5-3mm spacing—already within the "optimal" range identified. What changes to current technology are actually being proposed?

We thank the reviewer suggestion, this has been addressed in the clinical implications section:

Optimal MEM configurations may help to improve the accuracy of mapping procedures, leading to more targeted and effective ablation therapies. Proximity of electrodes to the reentrant path, in terms of contact between the electrode and the atrial wall, are crucial for visualizing AF reentrant drivers, so this may imply changes to the current mapping protocols and devices to ensure the best contact [32]. Current high-density mapping catheters [2, 33] may need to integrate unipolar and omnipolar mapping approaches during AF to enhance the detection of circuits sustaining AF.

Our findings advocate for a strategic rather than purely density-driven approach to AF mapping. Given that driver locations are patient-specific, initial wide-area panoramic mapping is necessary to identify regions potentially driving AF, such as with high dominant frequency or dispersed EGMs. Subsequent high-density interrogation of these areas should prioritize signal quality and catheter orientation, potentially utilizing unipolar and omnipolar integration to better detect localized reentry. Refinement may require iterative remapping with fine adjustments to catheter position and angle to account for local myofiber architecture. As microanatomic reentry cannot be visualized during sinus rhythm, this methodology is specifically intended for use during AF to improve the interpretation of suspected substrate. Consequently, clinical success depends not only on electrode density and contact but on a systematic workflow that transitions from broad regional identification to focused, multi-orientational remapping. Incorporating these insights into clinical practice could enhance perAF ablation success rates while potentially reducing recurrence and complications associated with AF ablation.

• Contact force: The dramatic difference between 0.25mm and 1mm contact seems arbitary and dependent on parameter selection

We apologize for the lack of clarity on this point. This has now been addresed in the limitations section:

Electrode contact with the atrial wall was only evaluated for binary surrogates of good (0.25 mm) and bad (1 mm) contact, but further contact configurations should be evaluated. Moreover, other potential effects of the anatomical conformation should be investigated, such as the alignment of the electrode and myobundle, curved surfaces of electrode and/or atrial wall, and anatomical irregularities in the atrial wall.

• Omnipolar findings: The conclusion that omnipolar offers "no advantage" at 3-6mm spacing is essentially dependent on the subjective way the authors present their data.

We apologize that the reviewer made this interpretation, which is not what we intended. In fact, the conclusions of the initial version of the manuscript describe omnipolar as being better for certain circumstances. However, we have rewritten that section to avoid confusion:

> *Omnipolar catheter configurations showed to be equivalent to unipolar mapping for dense MEMs (3–6 mm spacing) but exhibited significantly better identification rates at larger spacings (9 mm).*

Discussion:

• The limitations section is too brief given the significant constraints

In the new version of the manuscript, this section has been considerably lengthened, following the reviewers' advice. It now reads as follows:

> *While this study provides valuable insights into optimal MEM configurations for mapping AF drivers, certain limitations must be acknowledged. Simulations may not fully capture the complexities of in vivo human AF, where factors such as region-specific myofibers heterogeneity and fibrosis distribution, and dynamic electrophysiological changes during AF could influence mapping accuracy. However, the impact of fiber direction and tissue types on the same specific computational model were already evaluated in previous studies [19, 31]. The effect of the far field in unipolar signals, including ventricular activity, has not been considered in the analysis.*
>
> *All simulations involved a micro-reentrant path, which allowed us to validate the MEM maps against an EGM-independent ground truth. However, the performance of this type of high-density mapping should also be evaluated in other scenarios without micro-reentries, such as endo-epicardial dissociation or fibrotically-provoked irregular propagation, in order to quantify the occurrence of false positives. Throughout the manuscript, a minimum resolution of 1 mm was considered, as it constitutes an idealized yet technically achievable scenario for clinical mapping and falls comfortably below commercially available resolutions. It should be noted, however, that sub-millimeter resolutions may offer further improvements in mapping accuracy. Electrode contact with the atrial wall was only evaluated for binary surrogates of good (0.25 mm) and bad (1 mm) contact, but further contact configurations should be evaluated. Moreover, other potential effects of the anatomical conformation should be investigated, such as the alignment of the electrode and myobundle, curved surfaces of electrode and/or atrial wall, and anatomical irregularities in the atrial wall.*
>
> *EGM artifacts caused by discontinuous depolarization at the simulation edge (edge effects) can also affect the EGM shape, and therefore the detection of micro-reentries, in a virtual environment. However, for this study, this effect was evaluated under pacing conditions, and the edge effect artifact was found to be an order of magnitude smaller than the EGM amplitude. This study focused on a specific 3D cellular model of AF reentry, providing a cycle length equivalent to persistent AF, which may limit the generalizability of the findings to other AF driver subtypes. Furthermore, the study relied on simulated electrograms rather than patient-derived data, which may not fully reflect the variability encountered in clinical practice.*

• No discussion of computational model validation

We thank the reviewer for the appropiate suggestion, this point has been addressed on the limitations section:

Simulations may not fully capture the complexities of in vivo human AF, where factors such as region-specific myofibers heterogeneity and fibrosis distribution, and dynamic electrophysiological changes during AF could influence mapping accuracy. However, the impact of fiber direction and tissue types on the same specific computational model were already evaluated in previous studies [19, 31]. The effect of the far field in unipolar signals, including ventricular activity, has not been considered in the analysis.

• Translational pathway to actual catheter design is missing

This point has been addressed in the clinical implication and conclusion's sections:

Optimal MEM configurations may help to improve the accuracy of mapping procedures, leading to more targeted and effective ablation therapies. Proximity of electrodes to the reentrant path, in terms of contact between the electrode and the atrial wall, are crucial for visualizing AF reentrant drivers, so this may imply changes to the current mapping protocols and devices to ensure the best contact [32]. Current high-density mapping catheters [2, 33] may need to integrate unipolar and omnipolar mapping approaches during AF to enhance the detection of circuits sustaining AF.

…

Mapping was hindered by narrow micro-reentrant tracks, though reentrant mapping still detected tracks much thinner than the electrode spacing. Combining unipolar and omnipolar mapping in dense MEM configurations (3–6 mm spacing) may be the most effective strategy for identifying micro-anatomical reentrant paths. This approach could enhance the success rates of perAF ablation procedures by enabling more precise localization of arrhythmogenic circuits.

Add blinded assessment of LAT maps; Perform sensitivity analysis on detection threshold; Discuss topological principles;

We don't quite understand this comment, but in any case, we understand that these are topics that have been covered in previous comments.

CONCLUSION

This is technically sound computational work that addresses an important question about optimal mapping configurations. However, the clinical premise rests on a contested hypothesis with limited independent validation, the primary endpoint is subjective, and the practical implications remain unclear. Substantial revision is needed to provide balanced context and objective validation before this work can meaningfully inform clinical practice.

We hope that the changes made to the manuscript have been sufficient to improve it. In addition to the changes suggested by this reviewer, other substantial changes have also been included, such as additional simulation scenarios under other substrate conditions (Fig. 7), which together we believe have considerably improved the manuscript.

1. Tzeis S, Gerstenfeld EP, Kalman J, Saad EB, Sepehri Shamloo A, Andrade JG, Barbhaiya CR, Baykaner T, Boveda S, Calkins H, et al. 2024 European Heart Rhythm Association/Heart Rhythm Society/Asia Pacific Heart Rhythm Society/Latin American Heart Rhythm Society expert consensus statement on catheter and surgical ablation of atrial fibrillation. Europace. 2024;26. doi: 10.1093/europace/euae043

2. Duytschaever M, Van den Abeele R, Carlier N, Bezerra AS, Verstraeten B, Lootens S, Desplenter K, Okenov A, Nezlobinsky T, Shah D, et al. Atrial Topology for a Unified Understanding of Typical and Atypical Flutter. Circulation: Arrhythmia and Electrophysiology. 2024;17:e013102. doi: 10.1161/CIRCEP.124.013102

3. Vandersickel N, Hendrickx S, Van den Abeele R, Verstraeten B. Impact of topology on the number of loops during macro-re-entrant atrial tachycardia. Eur Heart J. 2024:ehae032. doi: 10.1093/eurheartj/ehae032

4. Arno L, Kabus D, Dierckx H. Strings, branes and twistons: topological analysis of phase defects in excitable media such as the heart. arXiv preprint arXiv:240102571. 2024.

5. Arno L, Kabus D, Dierckx H. Analysis of complex excitation patterns using Feynman-like diagrams. Scientific Reports. 2024;14:28962.

Reviewer #3

This original study applies computational models of atrial tissue to explore electrode configurations needed to map reentrant circuits during atrial fibrillation (AF). The results highlight a necessity of high-density mapping to detect micro-reentrant circuits and suggest practical ways for their mapping in clinical AF. Although the need to high-density mapping is intuitive, the paper provides a useful quantification of the optimal electrode spacing. The paper is generally well written, but can still benefit from some clarifications, particularly when it comes to methodology and practical value of results.

1) Introduction should make a stronger case for re-entry being a primary mechanism of AF (many clinicians would disagree), and also provide an overview of other hypothesis for AF mechanisms.

We thank the reviewer for their comments. We propose micro-reentrant path as a possible mechanism underlying AF perpetuation, but complementary to other known mechanisms such as multiple waves, endo-epicardial dissociation, functional reentries, and many others. We have focused on micro-reentrants because their applicability is limited by mapping techniques, which can be investigated *in silico*. The introduction has been revised to address these points and now reads as follows:

> *Primary theories regarding the maintenance mechanisms of persistent AF (perAF) revolve around either multi-wavelets propagating across the atria or localized AF drivers, electrical phenomena critical to AF perpetuation [2]. Posteriorly, AF has been defended to be sustained by drivers characterized by rapid reentrant electrical activity that spreads throughout the atria and can be temporally stable or unstable [3]. Targeted ablation of AF drivers reduces the ability to sustain AF and has shown promise in improving treatment efficacy and long-term outcomes compared to conventional pulmonary vein isolation [4-5]. However, guided ablation strategies going beyond pulmonary vein isolation currently lack sufficient scientific evidence to be recommended in clinical practice [6]. Accurately identifying patient-specific AF drivers poses a challenge due to the inadequate visualization of potential intramural reentrant drivers using surface-only multi-electrode mapping (MEM) [7]. This limitation has resulted in conflicting clinical mapping results and outcomes in AF driver ablation trials [2, 8].*

2) Methods should greatly benefit from more detailed description of multiple aspects of modelling:

- It is unclear how atrial bundles were modelled: sentence "Based on the 3D structure of AF driver reentrant track revealed by ex-vivo human heart 3D imaging…" makes little sense, and Fig. 1 it refers to is not too helpful as it shows functional, rather than structural variables;

We apologize for the lack of clarity on this important point. The description of the anatomical model used for the manuscript has been extended (Materials and Methods, Section *Biophysical simulation of a micro-anatomical reentry*), and we included a new Figure 7 in which this structural model is better shown (see Fig. 7 two comments below).

> *A 3D model of an atrial slab of tissue, including an insulated myobundle (336,241 nodes, 2,017,775 tetrahedral, 267 ± 53 µm inter-node distance) was used to simulate an AF pattern sustained by a micro-anatomical reentry. Based on prior ex vivo human studies of atrial driver regions [7, 9, 11-13], we constructed a simplified but representative human atrial driver-region geometry rather than a full chamber-specific reconstruction of either the right atrium (RA) or left atrium (LA). The model was designed to capture the essential 3D structural configuration shared by human atrial driver substrates, including a sub-endocardial, laterally-insulated myobundle reproducing the essential common micro-*

anatomic reentrant track configuration described in prior structural-functional studies. This reduced geometry was selected to preserve the key substrate features relevant to both RA and LA driver regions while allowing controlled evaluation of how electrode spacing, orientation, and wall contact influence MEM-based detection of the reentrant track. Based on the 3D structure of AF driver reentrant track revealed by ex vivo human heart 3D imaging [9], we constructed a 3D anatomical model consisting of a wedge of atrial wall tissue (30 × 30 × 4 mm) with a sub-endocardial, laterally-insulated myobundle (15 × 2.5 × 1.5 mm) (Figure 1), embedded in the atrial wall tissue.

- Was there a single bundle, or fibre orientation was modelled throughout the tissue? In the latter case, more details should be provided, in the former case it's unclear why most of the tissue was isotropic and how the lack of tissue anisotropy would affect the electrograms?

We apologize for the lack of clarity on this point. All tissue was considered anisotropic, both atrial wall and myobundle, with the fiber orientation aligned with the myobundle orientation. This has been described in more detail in the Materials and Methods section (Section *Biophysical simulation of a micro-anatomical reentry)*:

Anisotropy of the cardiac tissue was applied varying the longitudinal and transversal diffusion (1:1.36 ratio in conduction velocity) [17-18] respect to the fiber direction, which was constant and aligned with the laterally-insulated myobundle orientation. Diffusion tensor was calculated with its maximal component D_{long} aligned with the fiber direction and the minimal D_{trans}, perpendicular to the fiber direction. Anisotropy ratios of D_{long}/D_{trans} were applied for both atrial wall and myobundle region according to previous studies [19].

- How tissue conductivies were validated: in particular, did the resulting conduction velocities correspond to clinical measurements; what was a rationale for 40% reduction in the bundle?

The reduction of the 40% was included to adapt the myobundle CV to the slow propagation experimentally identified in structures such as the pectinate muscles. This description can be read in Materials and Methods section (Section *Biophysical simulation of a micro-anatomical reentry):*

Heterogeneity in the electrophysiological properties of sub-endocardial laterally-insulated myobundle with respect to the atrial myocardium was introduced as reduced tissue conductivity (-40% respect to atrial wall) in order to simulate slow-path conduction identified in experimental studies [16].

The relatively slow CV used on the biophysical model were are discussed in the Discussion section (Section *Human AF maintenance by reentrant driver mechanisms*):

Evidences of AF maintenance due to atrial structures such as the pectinate bundles have also been reported using MEMs [16], including similar conditions (1.5 cm track, conduction velocity of 13 to 47 cm/s) to the ones simulated here. The relatively slow conduction velocities used in the micro-reentrant track were chosen to reproduce bundle-dependent slow conduction reported in prior experimental studies of atrial reentry. Similar conditions have been described for pectinate muscle-related intra-atrial reentry, with conduction velocities ranging from 13 to 47 cm/s [16], and in our ex vivo human optical mapping studies, where localized AF driver tracks of comparable dimensions showed conduction velocities around the reentrant circuit of 33 ± 14 cm/s [24] and micro-anatomic reentrant tracks measuring approximately 15.4 × 6.0 mm were directly identified in human atria [9].

Moreover, to evaluate the impact of the slow condution on the micro-reentrant path, we have introduced new simulations in which the conduction velocity of the myobundle is greater than

the baseline simulation and equal to the CV of the atrial wall (Figure 7). This has been added to the Results section (Section *Effect of substrate condition on micro-reentrant path identification*)

Finally, micro-reentry detection was evaluated in a simulation in which the conduction velocities of the micro-reentrant track and the atrial wall were aligned at approximately 21 cm/s (Figure 7D). Overall, this case showed a slight improvement over the baseline case. For unipolar mapping, the results of automatic detection were similar or slightly better (~10%). In omnipolar mapping, notable improvements were observed for the 9-mm spacing, where detection rates increased from 50% to 80%, similar to bipolar mapping. Combined unipolar and omnipolar detection also improved slightly (~10%) for 3- and 6-mm spacings.

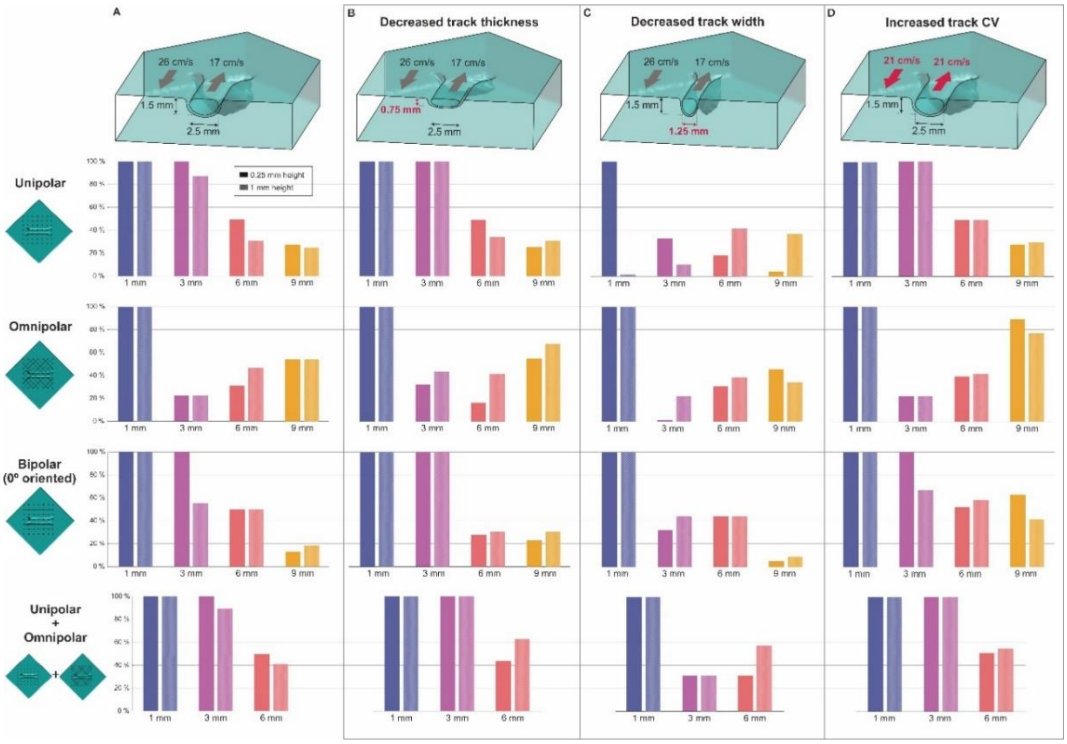

Figure 7. Micro-reentry detection under different substrate conditions. A) Baseline substrate. B) Micro-reentrant track with reduced thickness to 50% respect to baseline. C) Micro-reentrant track with reduced width to 50% respect to baseline. D) Micro-reentrant track with increased 19specto19, so atrial wall and micro-reentrant track conduction velocity are similar.

- Electrogram calculation section provides very 19spect information and can 19specto from a diagram to illustrate the electrode configurations or/and a table to summarise multiple configurations; besides, what was a rationale for choosing specific electrode radii, lengths and spacings?

We apologize for the lack of clarity on this point. Following the reviewer suggestion, we better described the electrode configuration on the Materials and Methods section (Section *Electrogram calculation*):

Simulated unipolar EGMs were calculated using cylindrical electrodes (0.5 mm radius, 1 mm length, similar to grid and basket-type 19specto19f catheters) positioned on a 2D plane above the endocardial 19specto, using two different distances in the normal direction (0.25, 1 mm) between these electrodes and endocardial wall where the myobundle is embedded. EGMs were calculated using Eq. (1) adding all effective transmembrane contributions over the entire model.

We also incuded a table (Table 1 in Section *Multi-electrode mapping*) with the distribution of the multi-electrode arrangements, included in the answer to the next comment.

- The following section should also aim to present the data in a more organized and justified matter: numerous numbers, such as inter-electrode distances, separations from the wall, displacements, etc all appear arbitrary and make the paper difficult to follow – especially when it ultimately come to the sentence "The simulations were divided into 254 unipolar, 292 bipolar and 110 omnipolar configurations; 328 configurations… where 20specto20f the numbers is lost.

We apologize for the lack of clarity in this 20specto. Following the reviewer concern, we included a table to describe the electrode configuration used in the manuscript, as well as a detailed description of the MEM configuration in the Material and Methods section (Section *Multi-electrode mapping*):

> *To evaluate the effect of the electrode distribution and location to properly map this micro-reentry, N=656 electrode configurations were used for mapping, described on Table 1. Different inter-electrode distances (1, 3, 6 and 9 mm) were evaluated, as well as 2 different separations respect to the atrial wall (0.25 mm and 1 mm). For each configuration, the multi-electrode (MEM) catheter, defined as an initial square grid of $N_x \times N_y$ electrodes, was systematically displaced relative to the micro-reentrant pathway. The displacement consisted of a 1 mm shift from the initial position along the two axes parallel to the atrial wall, generating horizontal, vertical, and combined offsets in both directions. When the grid was displaced, electrodes falling outside the atrial geometry were excluded from the analysis, resulting in reduced/rectangular $N_x \times N_y$ grid sizes in some configurations. The simulations were divided into 254 unipolar, 292 bipolar and 110 omnipolar configurations; 328 configurations for each separation (0.25 and 1 mm); and 8, 72, 288 and 288 for each inter-electrode distance (1, 3, 6 and 9 mm).*

> *Table 1. Electrode configuration. Number of simulations performed per electrode configuration. Size: unipolar electrodes per configuration ($N_x \times N_y$)*

Contact	Electrode spacing	Sizes $N_x \times N_y$	Electrode type: Unipolar	Bipolar 0º	Bipolar 90º	Omnipolar
0.25 mm	1 mm	21×21	N = 1	N = 1	N = 1	N = 1
	3 mm	7×7, 6×7, 6×6	N = 9	N = 9	N = 9	N = 9
	6 mm	4×4, 3×4, 3×3	N = 36	N = 36	N = 36	N = 36
	9 mm	3×3, 3×2, 2×2	N = 81	N = 27	N = 27	N = 9
1 mm	1 mm	21×21	N = 1	N = 1	N = 1	N = 1
	3 mm	7×7, 6×7, 6×6	N = 9	N = 9	N = 9	N = 9
	6 mm	4×4, 3×4, 3×3	N = 36	N = 36	N = 36	N = 36
	9 mm	3×3, 3×2, 2×2	N = 81	N = 27	N = 27	N = 9

- Importantly, there is no mention of how AF was initiated (fast pacing, S1-S2, etc) and whether the activity simulated could indeed be classified as persistent AF (any quantitative mertics)?

We apologize for missing this important point, it has been included in the Materials and Methods (Section *Biophysical simulation of a micro-anatomical reentry*):

The simulation was first stabilized by regular pacing from the atrial wall next to the myobundle exit site (CL=200ms, 10 beats), and the reentry was initiated by the induction of a conduction block in the myobundle exit site.

Regarding the AF classification, this has been included in the limitations section:

This study focused on a specific 3D cellular model of AF reentry, providing a cycle length equivalent to persistent AF, which may limit the generalizability of the findings to other AF driver subtypes.

3) Results are generally better organized, but could still benefit from more systematic presentation of numerous configurations (maybe a table) and more clarify in the figures: specifically, all figures should be labelled to clearly demonstrate directions of reentrant wave movement (arrows) /conduction blocks.

Again, we appreciate the reviewer suggestion. We included the Table 1, described in the two comments above comment. Additionally, we also included in all LAT maps, the automatically-detected reentrant path, with purple colors. Here we reproduce one of the main figures with the reentrant path:

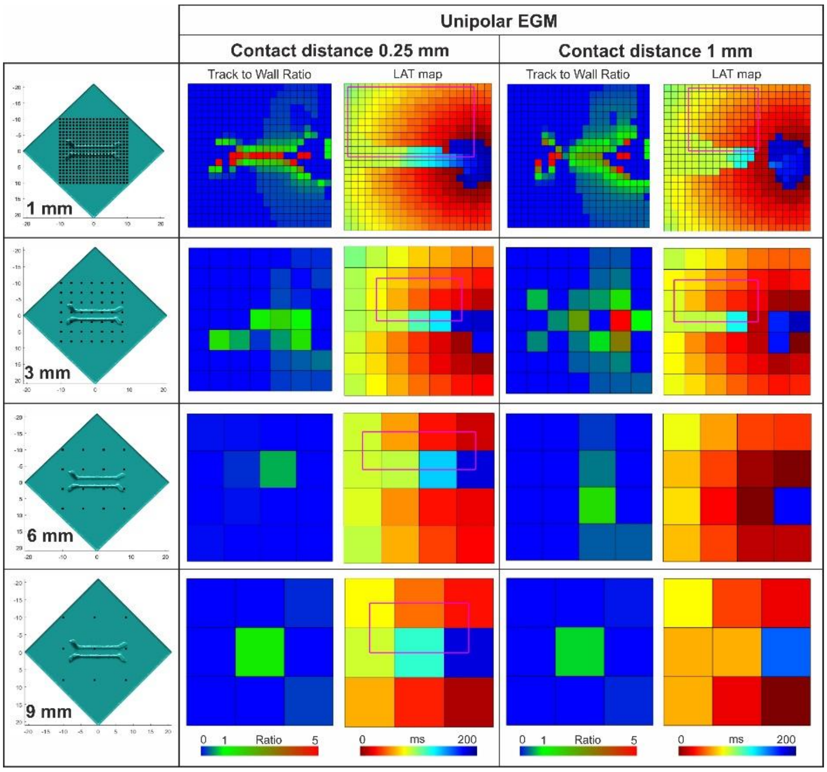

Figure 2. Unipolar EGM mapping of micro-anatomical reentry. Left panels illustrate the schematic EGM positions relative to the reentrant structure for each inter-electrode spacing (1, 3, 6, and 9 mm). Middle and right panels display the recorded reentry track-to-wall ratio and LAT maps, respectively, for each inter-electrode spacing (1, 3, 6, and 9 mm) and two EGM distances to the atrial wall (0.25 mm and 1 mm). Magenta rectangles on LAT maps represent the reentrant path identified, with the highest coverage of the cycle length.

4) Discussion/limitations should elaborate of potential effects of simplistic rectangular geometry: e.g., would the results change due to uneven alignment of the electrodes with curved atrial surfaces.

We thank the reviewer for the appropiate sugestion, this point has been discussed as follows:

> *Moreover, other potential effects of the anatomical conformation should be investigated, such as the alignment of the electrode and myobundle, curved surfaces of electrode and/or atrial wall, and anatomical irregularities in the atrial wall.*

Importantly, the Clinical implications section should offer a more practical guidance - it is intuitive that more dense electrodes provide better mapping, are there any other practical take-home messages for clinicians? For example, the paper states that "proximity of electrodes to the reentrant path" is crucial for detecting re-entries – but how can you move electrode closer to circuits before mapping, when you don't know where this circuits are? Another key question is, what would be a practical value of this in situation when majority of mappings are done in sinus rhythm, when there may be anatomical circuits but no reentrant activity? Any other practical considerations for clinicians to help them in AF mapping?

We thank the reviewer for the appropiate sugestion, this point has been discussed as follows:

> *Optimal MEM configurations may help to improve the accuracy of mapping procedures, leading to more targeted and effective ablation therapies. Proximity of electrodes to the reentrant path, in terms of contact between the electrode and the atrial wall, are crucial for visualizing AF reentrant drivers, so this may imply changes to the current mapping protocols and devices to ensure the best contact [32]. Current high-density mapping catheters [2, 33] may need to integrate unipolar and omnipolar mapping approaches during AF to enhance the detection of circuits sustaining AF.*
>
> *Our findings advocate for a strategic rather than purely density-driven approach to AF mapping. Given that driver locations are patient-specific, initial wide-area panoramic mapping is necessary to identify regions potentially driving AF, such as with high dominant frequency or dispersed EGMs. Subsequent high-density interrogation of these areas should prioritize signal quality and catheter orientation, potentially utilizing unipolar and omnipolar integration to better detect localized reentry. Refinement may require iterative remapping with fine adjustments to catheter position and angle to account for local myofiber architecture. As microanatomic reentry cannot be visualized during sinus rhythm, this methodology is specifically intended for use during AF to improve the interpretation of suspected substrate. Consequently, clinical success depends not only on electrode density and contact but on a systematic workflow that transitions from broad regional identification to focused, multi-orientational remapping. Incorporating these insights into clinical practice could enhance perAF ablation success rates while potentially reducing recurrence and complications associated with AF ablation.*
