# Supplementary material for: Catheter configuration for mapping micro-anatomic reentries sustaining atrial fibrillation: A simulation study
Source: PLoS Comput Biol. 2026 Jul 24;22(7):e1014493. doi: 10.1371/journal.pcbi.1014493 (PMC13399530; doi:10.1371/journal.pcbi.1014493)
Supplement: Peer review history

# Peer review: Decision Letter 0

**DOI:** 10.1371/journal.pcbi.1014493.r001

**Authors:** Feilim Mac Gabhann, Haibo Ni

2 Feb 2026

PCOMPBIOL-D-25-01546

Catheter Configuration for Mapping Micro-Anatomic Reentries sustaining Atrial Fibrillation: a simulation study

PLOS Computational Biology

Dear Dr. Miguel,

Thank you for submitting your manuscript to PLOS Computational Biology. After careful consideration, we feel that it has merit but does not fully meet PLOS Computational Biology's publication criteria as it currently stands. Therefore, we invite you to submit a revised version of the manuscript that addresses the points raised during the review process.

Please submit your revised manuscript by Apr 04 2026 11:59PM. If you will need more time than this to complete your revisions, please reply to this message or contact the journal office at ploscompbiol@plos.org. When you're ready to submit your revision, log on to https://www.editorialmanager.com/pcompbiol/ and select the 'Submissions Needing Revision' folder to locate your manuscript file.

Please include the following items when submitting your revised manuscript:

* A letter that responds to each point raised by the editor and reviewer(s). You should upload this letter as a separate file labeled 'Response to Reviewers'. This file does not need to include responses to formatting updates and technical items listed in the 'Journal Requirements' section below.

* A marked-up copy of your manuscript that highlights changes made to the original version. You should upload this as a separate file labeled 'Revised Manuscript with Track Changes'.

* An unmarked version of your revised paper without tracked changes. You should upload this as a separate file labeled 'Manuscript'.

If you would like to make changes to your financial disclosure, competing interests statement, or data availability statement, please make these updates within the submission form at the time of resubmission. Guidelines for resubmitting your figure files are available below the reviewer comments at the end of this letter

We look forward to receiving your revised manuscript.

Kind regards,

Haibo Ni

Academic Editor

PLOS Computational Biology

Feilim Mac Gabhann

Editor-in-Chief

PLOS Computational Biology

**Additional Editor Comments (if provided):**

The manuscript has been reviewed by three experts in the field of both experimental and computational electrophysiology. While the reviewers generally agreed upon the validity of the modeling approach, several issues have been noted, including the detailed methodology, practical use of the results, among many others.

**Journal Requirements:**

If the reviewer comments include a recommendation to cite specific previously published works, please review and evaluate these publications to determine whether they are relevant and should be cited. There is no requirement to cite these works unless the editor has indicated otherwise.

[Note: HTML markup is below. Please do not edit.]

**Reviewers' comments:**

Reviewer's Responses to Questions

**Comments to the Authors:**

**Please note here if the review is uploaded as an attachment.**

Reviewer #1: This paper computes electrograms over a cardiac muscular bridge. It examines

how electrode spacing affects the ability to detect the structure. The authors

examine the effect of spacing on several electrode types and conclude that a

certain resolution is required for its detection. The situation modelled is

very simple and does not capture the full physiological detail, but it may be

sufficient for the point that the authors are trying to make. The conclusions

reached by the authors are as expected. The paper is well organized and

logical. The English needs improvement in a few places. Concerns about the

paper are given below:

The authors need to describe how they processed signals for the omnipole and

used them. The authors treat the LATs obtained from such as being based on

bipoles, but they are derived from unipoles (DOI:10.1161/CIRCEP.116.004107) and

used to construct the virtual bipoles. Thus, LATs as derived from omnipoles

should never be worse than unipoles. Also, the authors are ignoring the

additional information obtained from the omnipole such as CV and direction.

Some of the parameters which influence the EGM seem arbitrary. For example, the

bridge conduction velocity is set lower than the wall, which will lower its EGM

amplitude. Also, the tissue thickness will affect the ratio of the signals. The

authors conclusions about electrode spacing distance also seem dependent on the

width of the bridge used. A thinner bridge would require finer sampling.

The authors do not specify a location for the tissue they are simulating other

than atrial. This is important due to the difference in structure between the

atria. The RA layer tends to be thinner with all fibers in the same direction,

and muscle bundles laid on top of that. The LA is thicker with two distinct

layers of differently oriented fibres, which will affect EGMs.

How do the authors get 336k nodes? With 0.267 mm edge lengths, I calculate

something closer to 191k nodes.

Was the myobundle placed on top of the slab or within it? Were the electrodes

in the same plane, or were electrodes over the myobundle displaced a further

1.5 mm in the normal direction.

What are the authors trying to say in lines 18-22. Please rephrase with

complete sentences. It is not clear.

A cycle length of 174 ms seems too long, as using the given conduction

velocities (17 and 26 cm/s), the time for a reentry would be about 145 ms. Do

the authors have references for these particular velocities? They are quite

low.

Did displacing the 1 mm grid by 1 mm do anything? Would it not have been better

to displace the grids by half of their sampling resolution?

Are edge effects seen in the EGMs, as the excitation/repolarization wavefronts hit the edges?

The authors could also comment on previous research looking at optimal electrode spacing.

Reviewer #2: PEER REVIEW: PCOMPBIOL-D-25-01546

Title: Catheter Configuration for Mapping Micro-Anatomic Reentries sustaining Atrial Fibrillation: a simulation study

SUMMARY

This computational study evaluates multi-electrode mapping (MEM) configurations for detecting micro-anatomic reentrant circuits that purportedly sustain atrial fibrillation. While the simulation methodology is straightforward and the technical analysis of electrode configurations is present, the paper has significant limitations regarding the clinical relevance of the substrate being modeled, selective citation of supporting evidence, and subjective outcome measures.

MAJOR CONCERNS

1. Limited Independent Evidence for Micro-Reentrant Driver Hypothesis

The paper's premise rests heavily on the concept that AF is maintained by intramural micro-anatomic reentrant circuits within fibrotically-insulated myobundles. However, the supporting evidence comes predominantly from the authors' own optical mapping studies [refs 6, 8, 9, 19].

Indeed, when evidence is considered in aggregate in conjunction with contemporary clinical practice as evidenced by the HRS/EHRA/APHRS current consensus statement there is a paucity of randomised trial evidence supporting the use of any form of driver based ablation;1 none of the lead authors of the contemporary clinical consensus publicly supports the use of driver based ablation.1

This is a fundamental and serious limitation of the manuscript.

2. Selective Presentation of Driver Ablation Efficacy

The introduction and discussion selectively cite studies supporting driver-targeted ablation [refs 4, 5] while omitting contradictory evidence. In practice, the interest of the clinical community in driver based ablation has declined.

3. Subjective Endpoint for Circuit Identification (Critical Limitation)

The study's primary outcome—"identification" of the micro-reentrant circuit—is defined as "reentrant LAT path in ≥4 electrodes representing >75% of the activation cycle" (page 7). This represents a subjective and arbitrary visual interpretation rather than an objective metric.

Major concerns:

• No quantitative validation against ground truth activation patterns

• No inter-observer reliability testing

• The 75% threshold appears arbitrary and unjustified

• The authors themselves determine which maps show "reentry"—potential for confirmation bias

4. Relationship to Topological Principles

The simulated reentrant circuits appear to follow topological balance principles described by Vandersickel et al. and others,2-5: The application of these principles provides a more meaningful pathway to achieve clinical relevance.

5. Unclear Clinical Significance

The clinical translation pathway remains vague:

• Electrode spacing: Current HD catheters (HD grid Advisor, Rhythmia) use 2.5-3mm spacing—already within the "optimal" range identified. What changes to current technology are actually being proposed?

• Contact force: The dramatic difference between 0.25mm and 1mm contact seems arbitary and dependent on parameter selection

• Omnipolar findings: The conclusion that omnipolar offers "no advantage" at 3-6mm spacing is essentially dependent on the subjective way the authors present their data.

Discussion:

• The limitations section is too brief given the significant constraints

• No discussion of computational model validation

• Translational pathway to actual catheter design is missing

Add blinded assessment of LAT maps; Perform sensitivity analysis on detection threshold; Discuss topological principles;

CONCLUSION

This is technically sound computational work that addresses an important question about optimal mapping configurations. However, the clinical premise rests on a contested hypothesis with limited independent validation, the primary endpoint is subjective, and the practical implications remain unclear. Substantial revision is needed to provide balanced context and objective validation before this work can meaningfully inform clinical practice.

1. Tzeis S, Gerstenfeld EP, Kalman J, Saad EB, Sepehri Shamloo A, Andrade JG, Barbhaiya CR, Baykaner T, Boveda S, Calkins H, et al. 2024 European Heart Rhythm Association/Heart Rhythm Society/Asia Pacific Heart Rhythm Society/Latin American Heart Rhythm Society expert consensus statement on catheter and surgical ablation of atrial fibrillation. Europace. 2024;26. doi: 10.1093/europace/euae043

2. Duytschaever M, Van den Abeele R, Carlier N, Bezerra AS, Verstraeten B, Lootens S, Desplenter K, Okenov A, Nezlobinsky T, Shah D, et al. Atrial Topology for a Unified Understanding of Typical and Atypical Flutter. Circulation: Arrhythmia and Electrophysiology. 2024;17:e013102. doi: 10.1161/CIRCEP.124.013102

3. Vandersickel N, Hendrickx S, Van den Abeele R, Verstraeten B. Impact of topology on the number of loops during macro-re-entrant atrial tachycardia. Eur Heart J. 2024:ehae032. doi: 10.1093/eurheartj/ehae032

4. Arno L, Kabus D, Dierckx H. Strings, branes and twistons: topological analysis of phase defects in excitable media such as the heart. arXiv preprint arXiv:240102571. 2024.

5. Arno L, Kabus D, Dierckx H. Analysis of complex excitation patterns using Feynman-like diagrams. Scientific Reports. 2024;14:28962.

Reviewer #3: This original study applies computational models of atrial tissue to explore electrode configurations needed to map reentrant circuits during atrial fibrillation (AF). The results highlight a necessity of high-density mapping to detect micro-reentrant circuits and suggest practical ways for their mapping in clinical AF. Although the need to high-density mapping is intuitive, the paper provides a useful quantification of the optimal electrode spacing. The paper is generally well written, but can still benefit from some clarifications, particularly when it comes to methodology and practical value of results.

1) Introduction should make a stronger case for re-entry being a primary mechanism of AF (many clinicians would disagree), and also provide an overview of other hypothesis for AF mechanisms.

2) Methods should greatly benefit from more detailed description of multiple aspects of modelling:

- It is unclear how atrial bundles were modelled: sentence “Based on the 3D structure of AF driver reentrant track revealed by ex-vivo human heart 3D imaging…” makes little sense, and Fig. 1 it refers to is not too helpful as it shows functional, rather than structural variables;

- Was there a single bundle, or fibre orientation was modelled throughout the tissue? In the latter case, more details should be provided, in the former case it’s unclear why most of the tissue was isotropic and how the lack of tissue anisotropy would affect the electrograms?

- How tissue conductivies were validated: in particular, did the resulting conduction velocities correspond to clinical measurements; what was a rationale for 40% reduction in the bundle?

- Electrogram calculation section provides very little information and can benefit from a diagram to illustrate the electrode configurations or/and a table to summarise multiple configurations; besides, what was a rationale for choosing specific electrode radii, lengths and spacings?

- The following section should also aim to present the data in a more organized and justified matter: numerous numbers, such as inter-electrode distances, separations from the wall, displacements, etc all appear arbitrary and make the paper difficult to follow – especially when it ultimately come to the sentence “The simulations were divided into 254 unipolar, 292 bipolar and 110 omnipolar configurations; 328 configurations… where origin of the numbers is lost.

- Importantly, there is no mention of how AF was initiated (fast pacing, S1-S2, etc) and whether the activity simulated could indeed be classified as persistent AF (any quantitative mertics)?

3) Results are generally better organized, but could still benefit from more systematic presentation of numerous configurations (maybe a table) and more clarify in the figures: specifically, all figures should be labelled to clearly demonstrate directions of reentrant wave movement (arrows) /conduction blocks.

4) Discussion/limitations should elaborate of potential effects of simplistic rectangular geometry: e.g., would the results change due to uneven alignment of the electrodes with curved atrial surfaces.

Importantly, the Clinical implications section should offer a more practical guidance - it is intuitive that more dense electrodes provide better mapping, are there any other practical take-home messages for clinicians? For example, the paper states that “proximity of electrodes to the reentrant path” is crucial for detecting re-entries – but how can you move electrode closer to circuits before mapping, when you don’t know where this circuits are? Another key question is, what would be a practical value of this in situation when majority of mappings are done in sinus rhythm, when there may be anatomical circuits but no reentrant activity? Any other practical considerations for clinicians to help them in AF mapping?

**********

**Have the authors made all data and (if applicable) computational code underlying the findings in their manuscript fully available?**

The PLOS Data policy requires authors to make all data and code underlying the findings described in their manuscript fully available without restriction, with rare exception (please refer to the Data Availability Statement in the manuscript PDF file). The data and code should be provided as part of the manuscript or its supporting information, or deposited to a public repository. For example, in addition to summary statistics, the data points behind means, medians and variance measures should be available. If there are restrictions on publicly sharing data or code —e.g. participant privacy or use of data from a third party—those must be specified.

Reviewer #1: Yes

Reviewer #2: Yes

Reviewer #3: Yes

**********

PLOS authors have the option to publish the peer review history of their article (what does this mean?). If published, this will include your full peer review and any attached files.

If you choose “no”, your identity will remain anonymous but your review may still be made public.

**Do you want your identity to be public for this peer review?**  For information about this choice, including consent withdrawal, please see our Privacy Policy.

Reviewer #1: No

Reviewer #2: No

Reviewer #3: No

[NOTE: If reviewer comments were submitted as an attachment file, they will be attached to this email and accessible via the submission site. Please log into your account, locate the manuscript record, and check for the action link "View Attachments". If this link does not appear, there are no attachment files.]

**Figure resubmission:**

While revising your submission, we strongly recommend that you use PLOS’s NAAS tool (https://ngplosjournals.pagemajik.ai/artanalysis) to test your figure files. NAAS can convert your figure files to the TIFF file type and meet basic requirements (such as print size, resolution), or provide you with a report on issues that do not meet our requirements and that NAAS cannot fix.

After uploading your figures to PLOS’s NAAS tool - https://ngplosjournals.pagemajik.ai/artanalysis, NAAS will process the files provided and display the results in the "Uploaded Files" section of the page as the processing is complete. If the uploaded figures meet our requirements (or NAAS is able to fix the files to meet our requirements), the figure will be marked as "fixed" above. If NAAS is unable to fix the files, a red "failed" label will appear above. When NAAS has confirmed that the figure files meet our requirements, please download the file via the download option, and include these NAAS processed figure files when submitting your revised manuscript.

**Reproducibility:**

To enhance the reproducibility of your results, we recommend that authors of applicable studies deposit laboratory protocols in protocols.io, where a protocol can be assigned its own identifier (DOI) such that it can be cited independently in the future. Additionally, PLOS ONE offers an option to publish peer-reviewed clinical study protocols. Read more information on sharing protocols at https://plos.org/protocols?utm_medium=editorial-email&utm_source=authorletters&utm_campaign=protocols

# Author comment: Author response to Decision Letter 1

**DOI:** 10.1371/journal.pcbi.1014493.r002

4 May 2026

Attachment Submitted filename: 260504_Reviewer response.pdf

# Peer review: Decision Letter 1

**DOI:** 10.1371/journal.pcbi.1014493.r003

**Authors:** Feilim Mac Gabhann, Haibo Ni, Feilim Mac Gabhann, Haibo Ni

24 Jun 2026

Dear Dr. Miguel,

We are pleased to inform you that your manuscript 'Catheter Configuration for Mapping Micro-Anatomic Reentries sustaining Atrial Fibrillation: a simulation study' has been provisionally accepted for publication in PLOS Computational Biology.

Before your manuscript can be formally accepted you will need to complete some formatting changes, which you will receive in a follow up email. A member of our team will be in touch with a set of requests.

Please note that your manuscript will not be scheduled for publication until you have made the required changes, so a swift response is appreciated.

IMPORTANT: The editorial review process is now complete. PLOS will only permit corrections to spelling, formatting or significant scientific errors from this point onwards. Requests for major changes, or any which affect the scientific understanding of your work, will cause delays to the publication date of your manuscript.

Should you, your institution's press office or the journal office choose to press release your paper, you will automatically be opted out of early publication. We ask that you notify us now if you or your institution is planning to press release the article. All press must be co-ordinated with PLOS.

Thank you again for supporting Open Access publishing; we are looking forward to publishing your work in PLOS Computational Biology.

Best regards,

Haibo Ni

Academic Editor

PLOS Computational Biology

Feilim Mac Gabhann

Editor-in-Chief

PLOS Computational Biology

***********************************************************

All concerns have been addressed in the revised manuscript.

Reviewer's Responses to Questions

**Comments to the Authors:**

**Please note here if the review is uploaded as an attachment.**

Reviewer #1: The authors have satisfactorily responded to all of my reviews.

Reviewer #2: I accept the revisions made by the authors as technically sound, but I have residual concern that the pursuit of micro-drivers will not lead to meaningful change in AF treatment.

I would strongly urge them to consider them alternative hypotheses for atrial fibrillation in future work in order to make genuine impact on patient care.

Reviewer #3: Thank you for addressing all my comments, you did a great job.

**********

**Have the authors made all data and (if applicable) computational code underlying the findings in their manuscript fully available?**

The PLOS Data policy requires authors to make all data and code underlying the findings described in their manuscript fully available without restriction, with rare exception (please refer to the Data Availability Statement in the manuscript PDF file). The data and code should be provided as part of the manuscript or its supporting information, or deposited to a public repository. For example, in addition to summary statistics, the data points behind means, medians and variance measures should be available. If there are restrictions on publicly sharing data or code —e.g. participant privacy or use of data from a third party—those must be specified.

Reviewer #1: None

Reviewer #2: **No:**  I couldn't see code submission, but maybe this is in other files?

Reviewer #3: Yes

**********

PLOS authors have the option to publish the peer review history of their article (what does this mean?). If published, this will include your full peer review and any attached files.

If you choose “no”, your identity will remain anonymous but your review may still be made public.

**Do you want your identity to be public for this peer review?**  For information about this choice, including consent withdrawal, please see our Privacy Policy.

Reviewer #1: No

Reviewer #2: No

Reviewer #3: **Yes:**  Oleg Aslanidi

# Editor report: Acceptance letter

**DOI:** 10.1371/journal.pcbi.1014493.r004

**Authors:** Feilim Mac Gabhann, Haibo Ni, Feilim Mac Gabhann, Haibo Ni

PCOMPBIOL-D-25-01546R1

Catheter Configuration for Mapping Micro-Anatomic Reentries Sustaining Atrial Fibrillation: A Simulation Study

Dear Dr Miguel,

I am pleased to inform you that your manuscript has been formally accepted for publication in PLOS Computational Biology. Your manuscript is now with our production department and you will be notified of the publication date in due course.

The corresponding author will soon be receiving a typeset proof for review, to ensure errors have not been introduced during production. Please review the PDF proof of your manuscript carefully, as this is the last chance to correct any errors. Please note that major changes, or those which affect the scientific understanding of the work, will likely cause delays to the publication date of your manuscript.

Soon after your final files are uploaded, unless you have opted out, the early version of your manuscript will be published online. The date of the early version will be your article's publication date. The final article will be published to the same URL, and all versions of the paper will be accessible to readers.

For Research, Software, and Methods articles, you will receive an invoice from PLOS for your publication fee after your manuscript has reached the completed accept phase. If you receive an email requesting payment before acceptance or for any other service, this may be a phishing scheme. Learn how to identify phishing emails and protect your accounts at https://explore.plos.org/phishing.

Thank you again for supporting PLOS Computational Biology and open-access publishing. We are looking forward to publishing your work!

With kind regards,

Kannan R K Kuppusamy, B.TECH BIOTECHNOLOGY

PLOS Computational Biology | Carlyle House, Carlyle Road, Cambridge CB4 3DN | United Kingdom ploscompbiol@plos.org | Phone +44 (0) 1223-442824 | ploscompbiol.org | @PLOSCompBiol
